# Frequency, Stressfulness and Type of Ethically Challenging Situations Encountered by Veterinary Team Members During the COVID-19 Pandemic

**DOI:** 10.3389/fvets.2021.647108

**Published:** 2021-04-12

**Authors:** Anne Quain, Siobhan Mullan, Paul D. McGreevy, Michael P. Ward

**Affiliations:** ^1^Faculty of Science, Sydney School of Veterinary Science, University of Sydney, Camperdown, NSW, Australia; ^2^Bristol Veterinary School, University of Bristol, Langford, United Kingdom; ^3^University of College Dublin School of Veterinary Medicine, Dublin, Ireland

**Keywords:** COVID-19, veterinary ethics, surveys, biosecurity, moral stress, pandemic

## Abstract

Ethically challenging situations (ECS) are common in veterinary settings and can lead to moral stress. However, there is no published information about how a global pandemic affects the frequency and types of ECS encountered by veterinary team members. An online mixed methods survey was developed to determine the frequency, stressfulness and types of ECS experienced by veterinarians, animal health technicians and veterinary nurses since the advent of the global COVID-19 pandemic in March 2020. Responses from 540 veterinary team members from 22 countries were analyzed. With the advent of the COVID-19 pandemic, the median frequency of ECS encountered by respondents increased from several times per month to several times per week (Spearman Rank Correlation 0.619, *P* < 0.0001). The most common ECS (encountered at least several times per week) were: *challenging decisions about how to proceed when clients have limited finances* (64.4%), *conflict between personal well-being and professional role* (64.3%), *conflict between the interests of clients and the interests of their animals* (59.6%). These were followed by *challenging decisions about what counts as an essential veterinary service* (48.1%); *conflict between well-being of family/household members and professional role* (46.3%); and *challenging decisions about whether to perform non-contact veterinary visits* (46.3%). The most stressful ECS (reported to be very or maximally stressful) were: *conflicts between the interests of clients and the interests of their animals* (50.2%), *other* (42.9%), *conflicts between the interests of my employer and my own interests* (42.5%), *challenging decisions about how to proceed when clients have limited finances* (39.4%), *conflict between personal well-being and professional role* (38.0%), and *conflict between well-being of family/household members and professional role* (33.6%). Thematic analysis of free-text responses revealed biosecurity, client financial limitations, animal welfare, working conditions, and client relations as prominent themes. This is, to the best of our knowledge, the first study to describe the impacts of the pandemic on ECS experienced by veterinary teams globally. It identifies an increase in the frequency of ECS associated with the COVID-19 pandemic, and a number of stressors unique to the pandemic. We identified a number of resources and strategies that may help veterinary team members navigate ethical challenges that may emerge in their daily work, as well as in the context of global crises.

## Introduction

Under normal circumstances, ethically challenging situations (ECS) are commonly encountered in veterinary settings and can lead to moral stress. Previous surveys have identified the most common ECS as client financial limitations restricting treatment options (1–3), and euthanasia in general (2). However, the most stressful ECS include clients wishing to continue treatment despite an animal's poor quality of life (4), suspected animal abuse (2), and euthanasia requests from clients who have funds but are unwilling to pay for treatment (3). In a study of 889 North American veterinarians, most reported feeling conflicted over what care to provide, and over 70% reported that obstacles preventing provision of appropriate care caused them or other veterinary team members moderate to severe distress (5). In general, veterinarians did not feel that their training adequately prepared them to manage ECS (3–5).

The COVID-19 pandemic, described as the second transboundary mega-crisis to impact contemporary societies in the 21st century after the global financial crisis (6), has necessitated radical change to everyday behaviors and working practices in most countries. Veterinary teams around the world were required to adapt to social distancing, restrictions on the types of services offered, restriction of non-essential travel, and pressure to reconsider what counts as a valid veterinary-client-patient relationship (VCPR). In addition, the limited surge capacity of human health care systems means that some veterinary teams donated or were required to forfeit personal protective equipment (PPE), medical equipment such as ventilators (7) and even staff for human healthcare (8). Due to restrictions on movement and closures of non-essential services, veterinarians may have been required to cull animals, for example surplus livestock (9) or animals in research settings (10). In some cases, veterinary team members were forced to limit the range and volume of services provided, due to lack of staff, limited access to PPE, or restrictions impacting ancillary services such as diagnostic laboratories and suppliers of goods and services (11, 12). One study documented a reduction in mental well-being of equine veterinarians and veterinary nurses since the advent of the COVID-19 pandemic (13).

Key stakeholders in veterinary ECS have historically been considered to be the veterinarian or veterinary team member, the animal patient and the client (14). Because of the highly infectious nature of SARS-CoV-2, and limited surge capacity of healthcare systems, veterinary team members now had to consider a wider range of stakeholders, including family members, human health care providers, and the community at large. As stated by Singleton and colleagues, “in the veterinary sector SARS-CoV-2 has led to practitioners being faced with a daily struggle to balance their responsibility to preserve animal welfare with ensuring the continued health of the public, colleagues and their families” (15).

Understanding the types of ECS encountered by veterinary team members during an unprecedented global crisis can assist in preparing for and potentially circumventing such challenges in the future.

To the authors' knowledge there are no published data on the impact of a transboundary mega-crisis on the ECS faced by veterinary team members. To address this gap, we conducted a survey to determine (1) the frequency, stressfulness and types of ECS encountered by veterinary team members during a global pandemic and (2) veterinary team members' approaches to recent ECSs.

## Materials and Methods

### Survey

We developed a survey comprising 29 questions, presented in three sections (see Supplementary Table 1). In the first section, participants were asked how often they experienced any ECS prior to the advent of COVID-19. They were then asked to describe, in their own words, the most common and the most stressful ECS encountered since the advent of COVID-19, respectively. Following this, they were asked to rate the frequency of a list of different ECS that they may have encountered in their work during the pandemic. This list was drawn from previous surveys of ECS in veterinary settings (2–5), review of available literature on the veterinary sector and COVID-19 at the time (March-April 2020), and discussion with veterinary colleagues (mostly in Australia, Italy, New Zealand, the US and the UK), about ECS encountered.

In the second section, participants were asked to consider the most recent situation in which they felt significant difficulty determining the ethically right thing to do. They were asked to choose a situation that had run its course and were advised that the example could come from any aspect of patient care or any other kind of situation in their workplace. They were asked to answer the following closed-ended questions in relation to that nominated situation: the type of ECS (from the same list as above), who or what was their primary obligation in this situation, how stressful was the situation, which strategies or resources they employed in the face of this situation, how helpful those strategies or resources were, how they rated the acceptability of the eventual outcome, what (if any) barriers to achieving an acceptable outcome they encountered, and, in reflecting on the case, what additional types of assistance or resources they would have found useful.

In the final section, participants were asked 9 demographic questions, including their professional role, country of work, year of graduation, year of birth, gender, caseload, hours worked per week in their current role, whether they were taught ethics as part of the training toward their qualification, and whether they had undertaken any ethics training after gaining their qualification. Participants were also asked how confident they are in dealing with ECS in their workplace, and to what extent they are free to make and act on ethical decisions in their workplace. For each closed-ended question, participants could select “other” and provide a free-text response. The final question asked participants “is there anything else you would like to add about your experience with ethically challenging situations since the advent of COVID-19?” This question was included to act as a safety net, to facilitate identification of pertinent issues that were not addressed in the preceding questions (16). There were no restrictions on the length of answers.

Research Electronic Data Capture (REDCap) was the survey platform used. REDCap is a secure web application used for building and managing surveys, as well as data storage and export, hosted by the University of Sydney.

The survey was piloted by veterinarians and veterinary nurses from a variety of backgrounds (industry, companion animals, equine practice, wildlife, veterinary education). Questions were refined on the basis of feedback from these individuals. The study was approved by the University of Sydney Human Research Ethics Committee (project 2020/291).

### Recruitment and Consent

A three-pronged online recruitment strategy was employed to maximize the networking potential of the study team and professional networks, and to distribute survey invitations as widely as possible across geographic boundaries (17). First, survey invitations were placed on websites or in electronic newsletters of professional bodies, professional organizations and special interest groups. The organizations who shared or published the link are listed in Supplementary Table 2. Second, a link to the survey was shared on social networking sites including Facebook and Twitter, as well as on the blog of one of the authors (AQ). Followers of these pages were able to share the link if they wished to. Third, survey invitations were distributed to professional networks of the study team via email.

Respondents were encouraged to share the survey link with colleagues, a snowball sampling technique which is an efficient and valid approach for recruiting unknown populations in online surveys (17). Respondents were invited to participate on a voluntary basis. No incentives were offered.

To meet the inclusion criteria, respondents were required to be a veterinarian, animal health technician or veterinary nurse over the age of 18 years. Participation was open to all geographic locations from the period 13 May 2020 to 14 July 2020. The landing page of the survey was a participant information statement, providing detailed information about the purpose of the study, the estimated completion time (15–20 min), information about data storage and feedback, and assurance of the confidentiality and anonymity of responses. Submission of responses via REDCap indicated consent to participate. Data were stored on the physically and electronically secure, restricted-access University of Sydney server, which is routinely backed up and accessible only by the study team.

### Data Cleaning

Where respondents had selected “other” from the drop-down menu and subsequently specified a response already represented by an option in the drop-down menu, it was re-categorized as such. Only those responses which were not reflected in the drop-down menu were retained in the “other” category.

### Quantitative Data

Survey data from REDCap were downloaded into Microsoft® Excel® for Microsoft 365 MSO (16.0.13328.20262). Responses were organized into categories for the purpose of descriptive statistics. Summary statistics were calculated for the demographic variables and for the ECS variables. Likert-style data were plotted using stacked bar graphs.

For the question on the frequency of specified types of ECS encountered since the COVID-19 pandemic, the categories “several times per day,” “daily” and “several times per week” were combined into “at least weekly” in order to better visualize the patterns present in the data.

For the question on the stressfulness of specified types of ECS encountered since the COVID-19 pandemic, the categories “very stressful” and “maximally stressful” were combined, as were the categories “a little bit stressful” and “moderately stressful” in order to better visualize the patterns present in the data.

IBM SPSS version 24 was used for statistical analysis. Pre- and post-COVID ECS distributions were assessed for normality, and median scores were calculated. The correlation (Spearman rank, r_SP_) between respondents pre vs. post COVID-19 ECS was estimated. Differences between groups were tested using the chi square test for categorical variables. A two-sided *p* < 0.05 was considered significant.

### Thematic Analysis

Thematic analysis of free-text responses was performed as described by Braun and Clarke (18, 19). Briefly, one author (AQ) familiarized herself with the data by reading all free-text responses multiple times. Using NVivo® 12 Plus software (QSR International), open codes were applied to represent concepts described by respondents. Themes and subthemes were actively constructed through an iterative data process analysis. Responses could be coded under multiple themes. A random subset of data (10%) was re-coded by two members of the research team (AQ and SM) to ensure inter-coder agreement on themes and subthemes at a minimum level of 80% (20). The authors then discussed differences in their coding. Frequencies of themes and subthemes were measured (21). Quotations from respondents are identified by professional role.

## Results

In total, 551 respondents completed the survey and pressed the “Submit” button at the end of the survey indicating their consent to participate. Of these, two were test responses and 9 pressed submit without providing any answers to survey questions. Therefore, 540 responses were analyzed. With the exception of one respondent, who did not answer one question, all respondents completed all questions. Therefore, 540 responses were analyzed for all questions, with the exception of the questions asking respondents to specify the most recent type of ECS they had encountered (*n* = 539), year of birth (*n* = 528), and whether the respondent had anything else they would like to add (*n* = 173). Most respondents were female (*n* = 434, 80.4%) and worked as veterinarians (78.3%, *n* = 423). Most (68%, *n* = 367) worked in companion animal practice. Those who selected “other” listed their caseload as comprising consultancy, shelter veterinary services, conservation biology, policy and research, and goats only. Most respondents worked 31–40 (34.4%, *n* = 186) or 41–50 (30.6%, *n* = 165) hours per week (65%, *n* = 351) (see Table 1). The year of graduation (*n* = 540) ranged from 1958 to 2020, with a mean of 2004 (SD 11.510) and median of 2007. The year of birth (*n* = 528) ranged from 1926 to 2000, with a mean of 1979 (SD 11.911) and median of 1980.

**Table 1:** Frequency table for the demographic information on respondents to mixed methods survey on ethically challenging situations encountered by veterinarians, animal health technicians and veterinary nurses globally in the COVID-19 era in 2020 (*n* = 540).

**Demographic parameter**	**Category**	**Number**	**Percentage%**
Gender	Female	434	80.4
	Male	102	18.9
	Other	4	0.7
Role	Veterinarian	423	78.3
	Veterinary nurse	97	18.0
	Animal health technician	11	2.0
	Other animal health professional	9	1.7
Caseload	Companion animal practice clinical	367	68.0
	Mixed animal practice clinical	38	7.0
	Academia/teaching	34	6.3
	Zoo and/or wildlife practice clinical	27	5.0
	Equine practice clinical	13	2.4
	Exotic/unusual pet practice clinical	12	2.2
	Practice management	12	2.2
	Non-government organization	10	1.9
	Scientific research/laboratory animals	8	1.5
	Government	8	1.5
	Other	5	0.9
	Industry (e.g., pharmaceutical companies, food companies)	4	0.7
	No longer working as a veterinarian	1	0.2
Hours/week	0–10	21	3.9
	11–20	31	5.7
	21–30	64	11.9
	31–40	186	34.4
	41–50	165	30.6
	50+	73	13.5
Country	Australia	319	59.1
	United States of America	125	23.1
	Canada	26	4.8
	United Kingdom	25	4.6
	New Zealand	12	2.2
	Singapore	10	1.9
	Germany	6	1.1
	China	4	0.7
	Netherlands	3	0.6
	Other^*^	13	2.4

* *Other included one respondent (0.2%) from each of the following countries: Austria, Belarus, Cambodia, Denmark, France, Hong Kong, Republic of Ireland, Jamaica, Lithuania, Mexico, Spain, Thailand, Zimbabwe. Percentages may not add to 100 due to rounding to one decimal place*.

**Figure 1:**
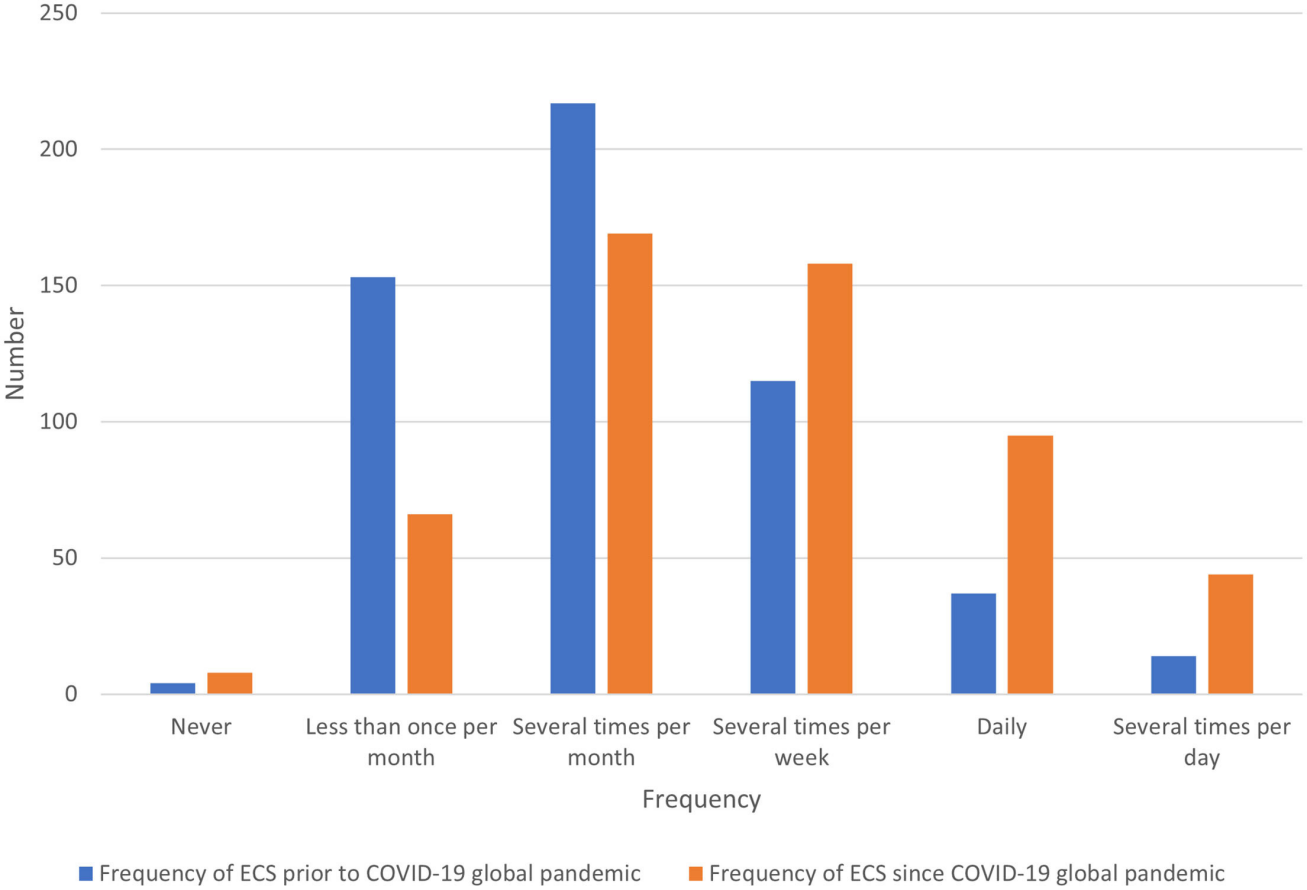
Bar chart for the median frequency of ethically challenging situations (ECS) encountered by veterinary teams prior to and since the advent of the COVID-19 global pandemic, based on the responses of 540 veterinarians, animal health technicians and veterinary nurses, surveyed between May and July in 2020.

The frequency of ECS encountered by veterinary team members increased following the advent of the pandemic (Figure 1). Prior to the pandemic, the median frequency with which veterinary team members reported encountering ECS was several times per month (interquartile range (IQR) once per month to several times per week). Following the advent of the COVID-19 pandemic, the median frequency increased to several times per week (IQR several times per month to at least once daily) (r_SP_ 0.619, *P* < 0.00001) (Table 2).

**Table 2:** Comparison of frequency of ethically challenging situations (ECS) encountered by veterinarians, animal health technicians and veterinary nurses prior to and since the advent of the COVID-19 global pandemic (*n* = 540).

		**Frequency of ECS since COVID-19 global pandemic**						
		**Less than once per month**	**Several times per month**	**Several times per week**	**Daily**	**Several times per day**	**Never**	**Total responses**
Frequency of ECS prior to COVID-19 global pandemic	Less than once per month	56	57	23	10	4	3	153
	Several times per month	8	108	74	22	4	1	217
	Several times per week	0	4	60	38	13	0	115
	Daily	1	0	0	25	11	0	37
	Several times per day	1	0	1	0	12	0	14
	Never	0	0	0	0	0	4	4
	Total responses	66	169	158	95	44	8	540

**Figure 2:**
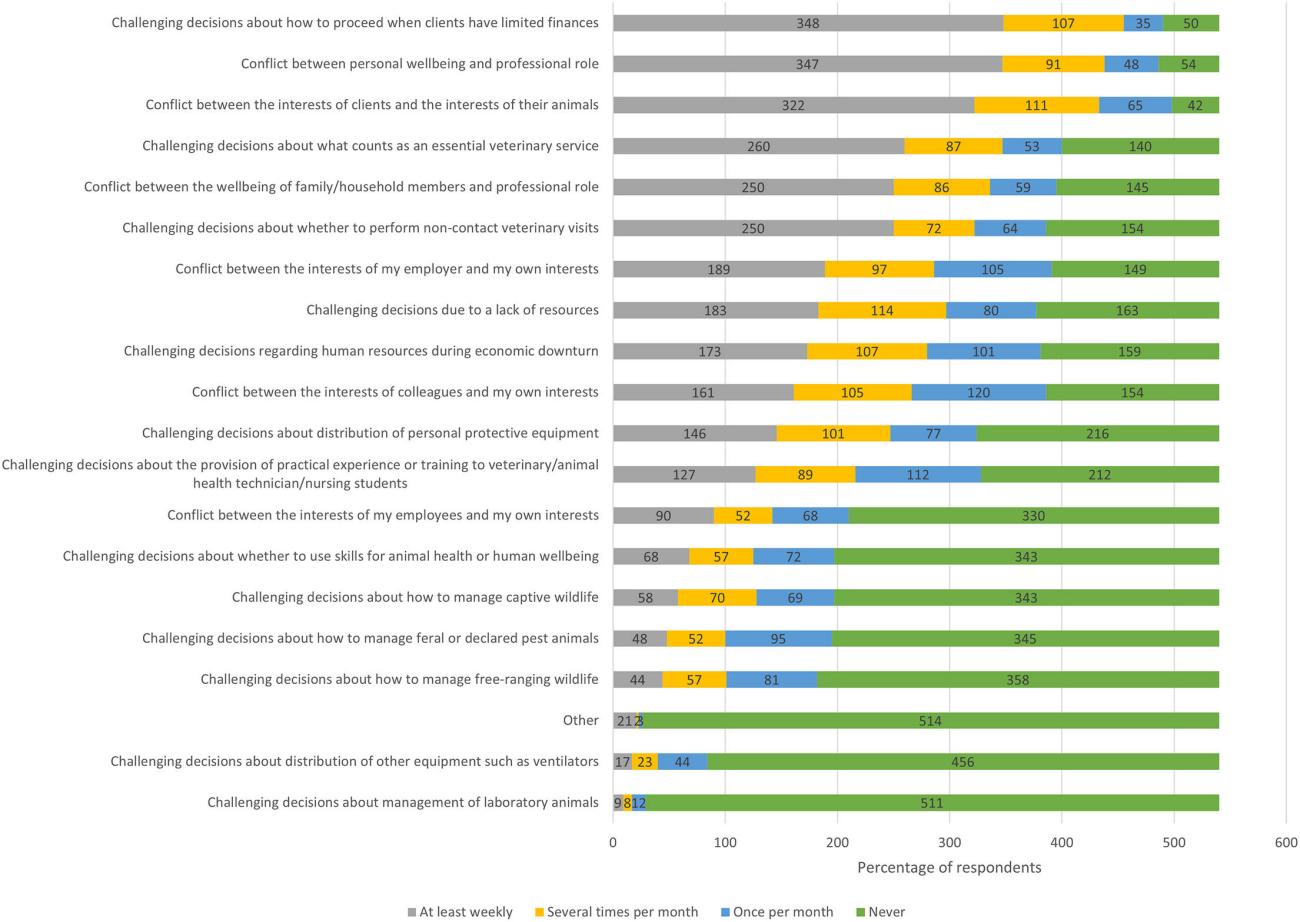
Stacked bar chart for the most commonly experienced ethically challenging situations encountered by veterinary team members since the advent of the COVID-19 pandemic, based on the responses of 540 based veterinarians, animal health technicians and veterinary nurses, surveyed between May and July in 2020.

The frequency at which respondents encountered different types of ECS is presented in Figure 2. The three most common ECS (encountered at least several times per week since the advent of the pandemic) were: *challenging decisions about how to proceed when clients have limited finances* (64.4%, *n* = 348), *conflict between personal well-being and professional role* (64.3%, *n* = 347), and *conflict between the interests of clients and the interests of their animals* (59.6%, *n* = 322). These were followed by *challenging decisions about what counts as an essential veterinary service* (48.1%, *n* = 260), *conflict between well-being of family/household members and professional role* (46.3%, *n* = 250), and *challenging decisions about whether to perform non-contact veterinary visits* (46.3%, *n* = 250).

Of the 22 respondents who additionally selected “other,” two did not provide an answer at all, and two simply provided contextual information which did not specify an ECS. The remaining 18 responses are included in Table 3.

**Table 3:** Other situations where decision-making may be ethically challenging, as described by participants.

**Role**	**Country**	**Ethically challenging situation as described by participant**
Veterinarian	Australia	Disagreements between colleagues. One person wants to do A and another wants to do B.
Veterinarian	Australia	Challenging decision about clients surrendering their animals out of failure to take care of them due to the imposed lockdowns.
Veterinarian	USA	Challenging to deal with staff who do not want to work or do not want to be effective at work or who just want a paycheck.
Veterinarian	Australia	Challenging decisions involving a difficult case, when clinicians do not collaborate and communicate effectively in order to achieve a better outcome.
Veterinary nurse	Australia	Seeing clients from other clinics that have been turned away from them as they are “too busy” to see them, when we are also double or triple booked. Some are from up to several hours drive away because everywhere is too busy.
Veterinarian	Germany	Home schooling an 8-year-old while working full time! how to deal with (euthanasia) home visits which I consider ethically essential.
Veterinarian	Australia	Allowing more than one owner into the clinic to be with their pet during euthanasia.
Veterinarian	Australia	Whether to wear PPE during home euthanasia visits. On the one hand I am wanting to protect the clients. I am not so worried about my own health. However, it feels impersonal. Also lack of physical contact with the owners at this time is challenging. Such as not being able to shake their hand or give them a hug.
Veterinarian	UK	Decisions around euthanasia which has to be carried out by others on site - mainly animal technicians - due to lack of ability to use animals in research due to lack of lab facilities (wastage).
Veterinarian	Germany	Hearing or reading pseudoscience, conspiracy theories, anti-vax-non-sense.there is always the question whether to keep my mouth shut or take the risk of a shitstorm;)
Veterinarian	USA	Information barrage from human healthcare, veterinary healthcare, federal, state, and university sources regarding epidemiology, legal, and policy changes.
Veterinarian	USA	We have had several owners and visitors wishing to enter the building, but our policy says they cannot.
Veterinarian	USA	Allowing DVMs to see enough cases to have reasonable income while limiting the schedule.
Veterinarian	USA	Provision of futile medical care to animals who are suffering.
Veterinarian	Australia	Challenging decisions about tolerance for risk of potential SARS-CoV-2 exposure to clients, carried by visiting veterinary team.
Veterinarian	Australia	I work as a vet in the live export industry. COVID-19 has put pressure on supply chains for both chilled meat and live animal exports. Now more than ever, it feels like I am stuck between two competing ideologies for and against the live export industry. People's opinions and heightened emotions are inhibiting sound decision making processes around balancing animal welfare with food security.
Veterinarian	USA	Lack of volunteers to perform duties; lack of donations to support operation.
Veterinarian	Australia	Difficulty with conflict with clients with regards to COVD protocols.

**Figure 3:**
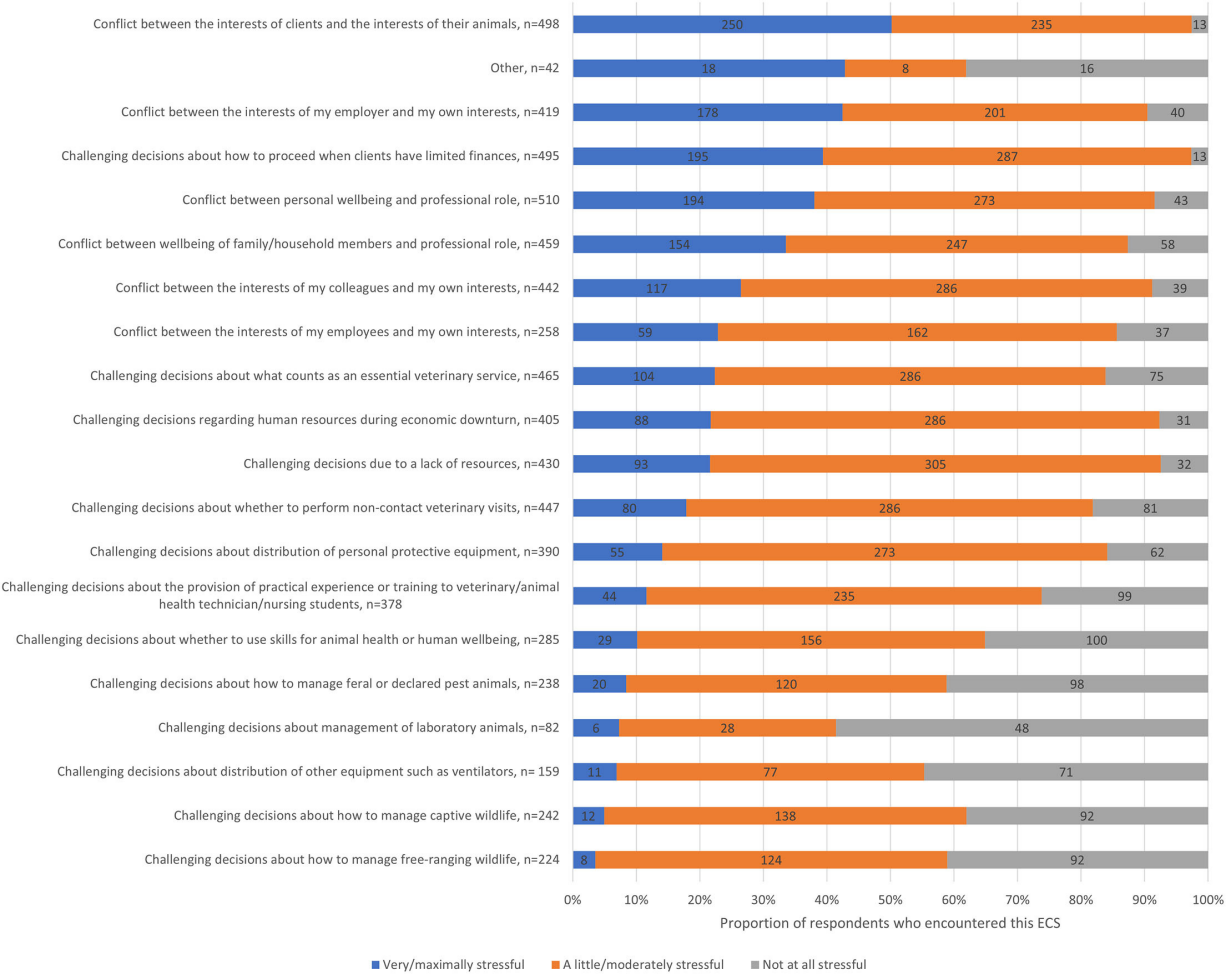
Stacked bar chart for the most stressful ethically challenging situations encountered by veterinary team members since the advent of the COVID-19 pandemic, based on the responses of 540 veterinarians, animal health technicians and veterinary nurses, surveyed between May and July in 2020.

The most stressful ECS was perceived to be *conflicts between the interests of clients and the interests of their animals*, reported as very or maximally stressful by 50.2% (*n* = 250) of the 498 respondents who had encountered it. More than one third of respondents reported the following ECS to be very stressful or maximally stressful (Figure 3): *other* (42.9%, *n* = 18/42), *conflicts between the interests of my employer and my own interests* (42.5%, *n* = 178/419), *challenging decisions about how to proceed when clients have limited finances* (39.4%, *n* = 195/495), *conflict between personal well-being and professional role* (38.0%, *n* = 194/510), and *conflict between well-being of family/household members and professional role* (33.6%, *N* = 154/459).

**Figure 4:**
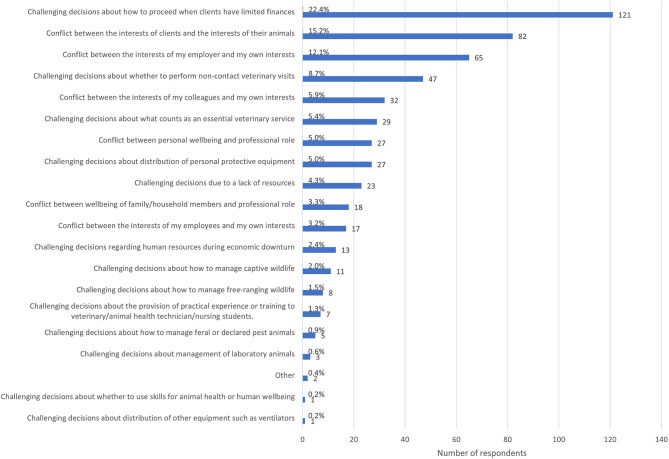
Bar chart of the frequency of the of the most recent type of ethically challenging situation encountered by veterinary team members since the advent of the COVID-19 pandemic, based on the responses of539 veterinarians, animal health technicians and veterinary nurses, surveyed between May and July in 2020.

When respondents were asked to consider the most recent situation in which they experienced significant difficulty deciding upon the right thing to do, 539 provided a response that specified the type of ECS (Figure 4). The most commonly selected types of ECS were *challenging decisions about how to proceed when clients have limited finances* (22.4, *n* = 121%), *conflict between the interests of clients and the interests of their animals* (15.2%, *n* = 82) and *conflict between the interests of my employer and my own interests* (12.1%, *n* = 65). Of the two respondents who selected other, one provided an irrelevant response, and one described having a disagreement with colleagues around case management.

When asked how stressful they found their most recent ECS, most respondents (54.2%) reported that this situation was either very stressful (37.2%, *n* = 201) or maximally stressful (17%, *n* = 92), another third of respondents indicated that it was moderately stressful (32.6%, *n* = 176) and 11.3% (*n* = 61) reported it was a little bit stressful. Only 1.9% (*n* = 10) reported that it was not stressful at all.

Almost half of respondents considered that ultimately, their primary obligation was to individual animal patients (480%, *n* = 259). The next most frequently selected categories were the community as a whole (13%, *n* = 70), other (12.6%, *n* = 68), my colleagues (10.2%, *n* = 55), individual clients (8.0%, *n* = 43), my employer (7.4%, *n* = 40), conservation of species (0.7%, *n* = 4) and the government (0.2%, *n* = 1).

Among the 68 response in the “other” category, respondents listed self (22.1%, *n* = 15), dual primary obligation (19.1%, *n* = 13) [humans and animals (*n* = 5), self and family (*n* = 3), workplace and regulator (*n* = 1), self and community (*n* = 1), self and colleagues (*n* = 1), family and work (*n* = 1), community and livestock industry (*n* = 1)], family (17.6%, *n* = 12), students/trainees/interns (11.8%, *n* = 8), business (4.4%, *n* = 3), the human animal bond (4.4%, *n* = 3), professional organizations (1.5%, *n* = 1), and the greater good (1.5%, *n* = 1). Additionally, two respondents (2.9%) stated that they were unsure of their primary obligation, two respondents who selected other did not provide any response (2.9%), and eight (11.8%) provided an irrelevant response.

**Figure 5:**
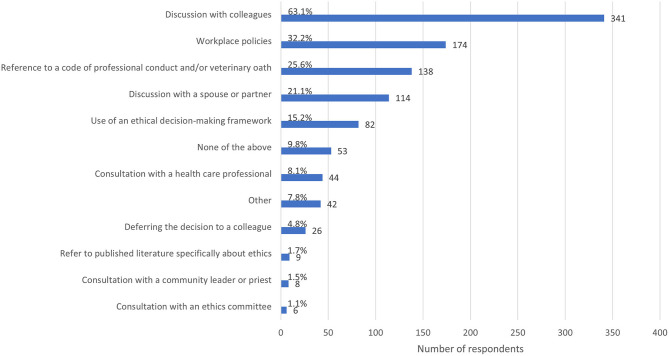
Bar chart of the frequency of the resources and strategies used by veterinary team members when faced with the most recent ethically challenging situation they have encountered, based on the responses of 540 veterinarians, animal health technicians and veterinary nurses, surveyed between May and July in 2020. Note that respondents could select multiple options.

The most commonly reported resource employed by respondents to help in the face of an ECS was discussion with colleagues (63.1%, *n* = 341), followed by workplace policies (32.2%, *n* = 174), reference to a professional code of conduct or veterinary oath (25.6%, *n* = 138), and discussion with a spouse or partner (21.1%, *n* = 114) (Figure 5).

While only 15.2% (*n* = 82) of respondents reported using an ethical framework, several respondents employed an ethical framework but did not recognize it as such. The most common ethical framework was utilitarianism, employing a cost-benefit analysis or harm reduction approach. For example,

“I used my knowledge of the clients, their known health status, our mutual trust, the need for euthanasia of their pet in the home environment and the refusal of a referral/emergency service to allow it.” (198, veterinarian, Australia)

Others appealed to a sense of what was “right” but did not elaborate on norms or rules they referred to in deliberation.

“I could only do what I felt was right for the client and the pet and what I could live with.” (222, veterinarian, US)

or

“What I felt was ultimately right although difficult/scary for me to do.” (270, veterinarian, Australia)

Other resources respondents used (7.8%, *n* = 42) were: application of unspecified problem solving skills (*n* = 5), reference to one's primary obligation (*n* = 4), risk minimization (*n* = 4), attending to personal well-being (yoga, meditation, mindfulness or exercise) (*n* = 3), communication with other stakeholders (*n* = 3), accessing and comparing guidelines from a number of organizations (*n* = 3), discussion with a friend or housemate (*n* = 2), a union, professional organization or regulatory body (*n* = 3), empathizing with one or more stakeholders (for example, trying to put oneself in the shoes of the owner) (*n* = 2), consulting a lawyer or legislation (*n* = 2), appealing to a sense of what feels “right (*n* = 2),” consulting a coach (*n* = 1), discussion with an insurer (*n* = 1), consultation with subject matter expert (*n* = 1), reference to scientific literature (*n* = 1), utilize principles of triage (*n* = 1), applying “common sense” (*n* = 1), utilizing one's own resources (unspecified) (*n* = 1), reference to past experience (*n* = 1) and a sense of common humanity (*n* = 1).

More than one third of respondents (35.9%, *n* = 194) found the resources and strategies they used somewhat helpful, while 30.4% (*n* = 164) found them helpful, 17.0% (*n* = 92) found them very helpful, and only 3.1% (*n* = 17) found them maximally helpful. In contrast, 6.3% (*n* = 34) found they were not helpful at all. In addition, 7.2% (*n* = 39) selected “not applicable” to this question.

Only 4.6% of respondents (*n* = 25) rated the outcome of the ECS as ideal, while 19.8% (*n* = 107) rated it as good, 46.1% (*n* = 249) rated it as an acceptable outcome which could be improved, 22.0% (*n* = 119) rated the outcome as uncertain, and 7.4% (*n* = 40) felt that the outcome was unacceptable.

**Figure 6:**
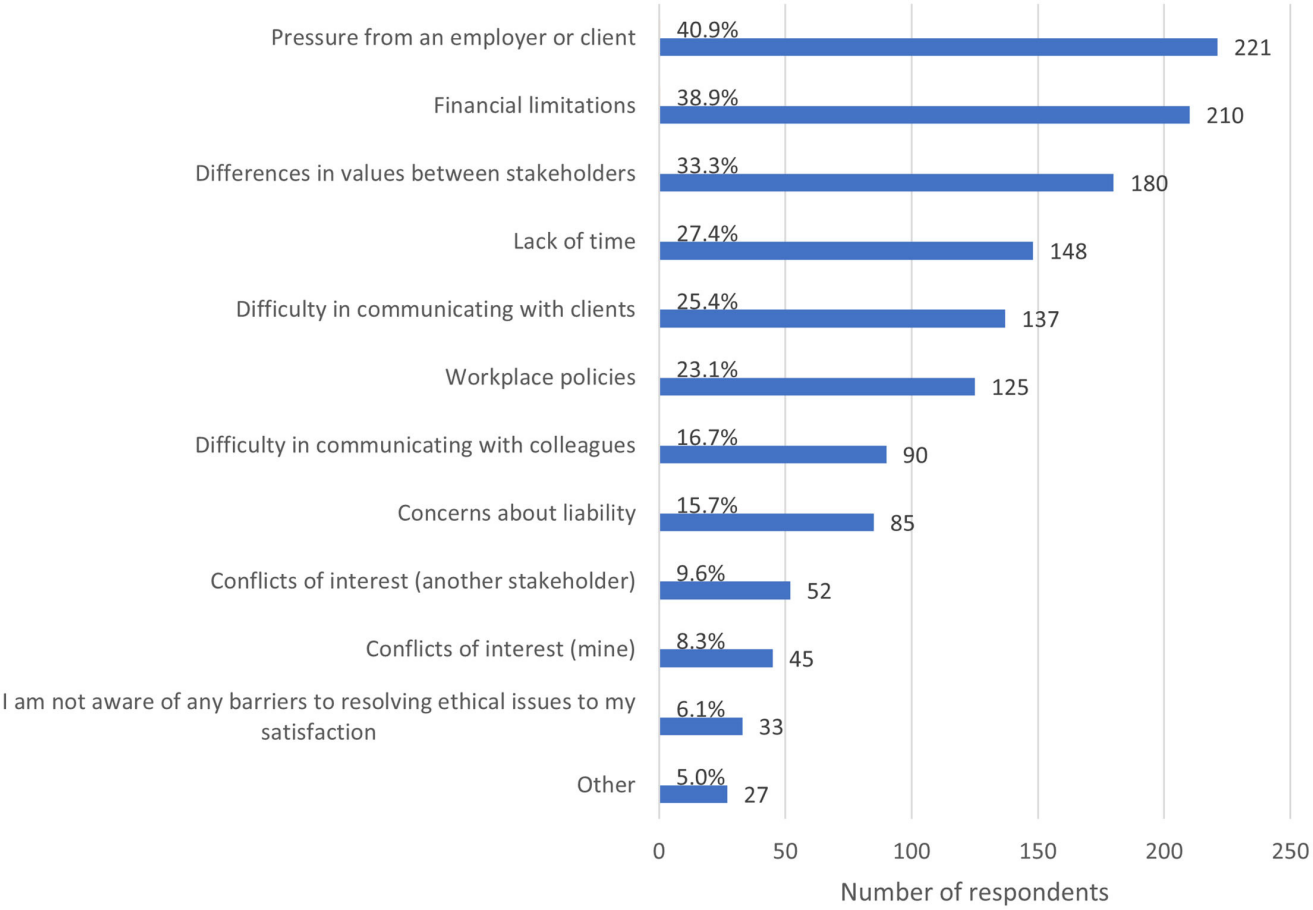
Bar chart of barriers to resolving the most recent ethically challenging situation encountered by veterinary team members, based on the responses of 540 veterinarians, animal health technicians and veterinary nurses, surveyed between May and July in 2020. Note that respondents could select multiple options.

The most common barrier to resolving an ECS to the respondent's satisfaction (Figure 6) was pressure from an employer or client (40.9%, *n* = 221), followed by financial limitations (38.9%, *n* = 210) and differences in values between stakeholders (33.3%, *n* = 180). Lack of time was a barrier in more than one quarter of cases (27.4%, *n* = 148). Only 6.1% (*n* = 33) of respondents reported not being aware of any barriers to resolving the ECS they described.

Other reported barriers included lack of personal resources (*n* = 4), rapidly changing recommendations, guidelines or restrictions (*n* = 3), lack of information or uncertainty (*n* = 3), lack of guidance from regulatory bodies, professional organizations or governments (*n* = 2), concerns for personal safety (*n* = 2), vulnerable clients (*n* = 2), government policies (*n* = 2), workplace culture (*n* = 2), shortage of human resources (*n* = 1), physical distancing (*n* = 1), a lack of services normally available (*n* = 1), unrealistic client expectations (*n* = 1), lack of support from a professional body (*n* = 1), racism (*n* = 1), and lack of resources in general (*n* = 1).

**Figure 7:**
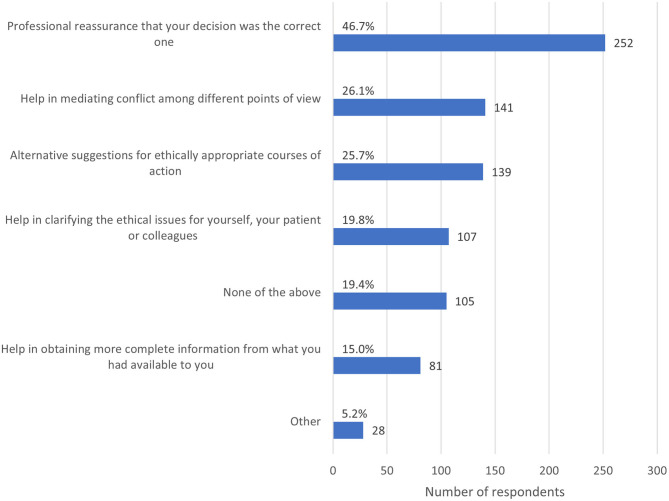
Bar chart of the types of assistance respondents felt would have been useful in resolving the most recent ethically challenging situation they encountered, based on the responses of 540 veterinarians, animal health technicians and veterinary nurses, surveyed between May and July in 2020. Note that respondents could select multiple options.

When asked to reflect on the ECS and consider which types of assistance they would have found useful, almost half (46.7%, *n* = 252) felt that professional reassurance that their decision was the correct one would have been useful (Figure 7). More than one quarter (26.1%, *n* = 141) reported that additional help in mediating conflict among different points of view would have been useful, and 25.7% (*n* = 139) reported that alternative suggestions for ethically appropriate courses of action would have been useful.

Among respondents who selected other, desired assistance for navigating ECS tended to fall into one of three major categories. The first category comprised practical support, and included human resources (*n* = 5), ability to provide financial support to clients (*n* = 2), more resources in general (*n* = 2), support in caring for one's family (*n* = 1), the support of an animal welfare organization (*n* = 1), more time (*n* = 1), financial support from the government (*n* = 1), communication skills (*n* = 1), flexibility from one's employer (*n* = 1) and pet insurance for the animal to fund diagnostics and treatment (*n* = 1). The second category referred to guidance, for example from guidelines, legislation or policy. This included the formal recognition of veterinary services as essential (*n* = 1), enforcement from a regulatory body (*n* = 1), effective government leadership (*n* = 1) and flexibility in policies (*n* = 1). The third category comprised ethics and decision making support, and included support for clients in making ethical decisions (*n* = 2), the ability to not worry about what others think (*n* = 1), the ability to discuss ECS within the workplace (*n* = 1), to be subpoenaed (*n* = 1) and “Black Lives Matter” (*n* = 1). One respondent said that they should not need other resources, and another did not specify what they intended when selecting other.

Most respondents (54.3%, *n* = 293) reported receiving some form of ethics training in obtaining the qualification for their current role, while 29.8% (*n* = 161) had none and 15.9% (*n* = 86) did not recall.

Following their qualification, 51.7% of respondents (*n* = 279), reported undertaking further training in ethics. Respondents could select multiple responses to this question. Just under one third (33.0%, *n* = 178) had undertaken continuing professional development (CPD) in ethics, 11.7% (*n* = 63) sat on an institutional ethics committee, 8.3% of respondents (*n* = 45) undertook university coursework in an ethics or bioethics degree, and 5.4% (*n* = 29) undertook another form of ethics training. These other forms of ethics training included private reading or discussion (*n* = 11), coursework for another a non-ethics or bioethics degree (*n* = 6), on the job training (*n* = 2), leadership training (*n* = 2), CPD that indirectly touches on ethics (*n* = 2), teaching ethics (*n* = 2), personal or professional experience (*n* = 2), membership of a professional organization (*n* = 1), and publishing in ethics (*n* = 1). Less than half of respondents 48.3% (*n* = 261) reported undertaking no post-qualification ethics training.

Most respondents were confident enough that they could get by (42.8%, *n* = 231) or reasonably confident (39.3%, *n* = 212) that they were able to deal with ECS in their workplace, while 3.3% (*n* = 18) reported that they couldn't be more confident. In contrast, 12.0% of respondents (*n* = 65) reported that they were under confident, and 2.6% (*n* = 14) were not confident at all in dealing with ECS.

The majority of respondents (52.4%, *n* = 283) reported that they were free to make and act on ethical decisions most of the time, compared to 20.4% (*n* = 110) sometimes, and 18.0% (*n* = 97) always. On the other hand, 7.4% (*n* = 40) were rarely and 1.9% (*n* = 10) were never free to make decisions in their workplace.

### Thematic Analysis

**Figure 8:**
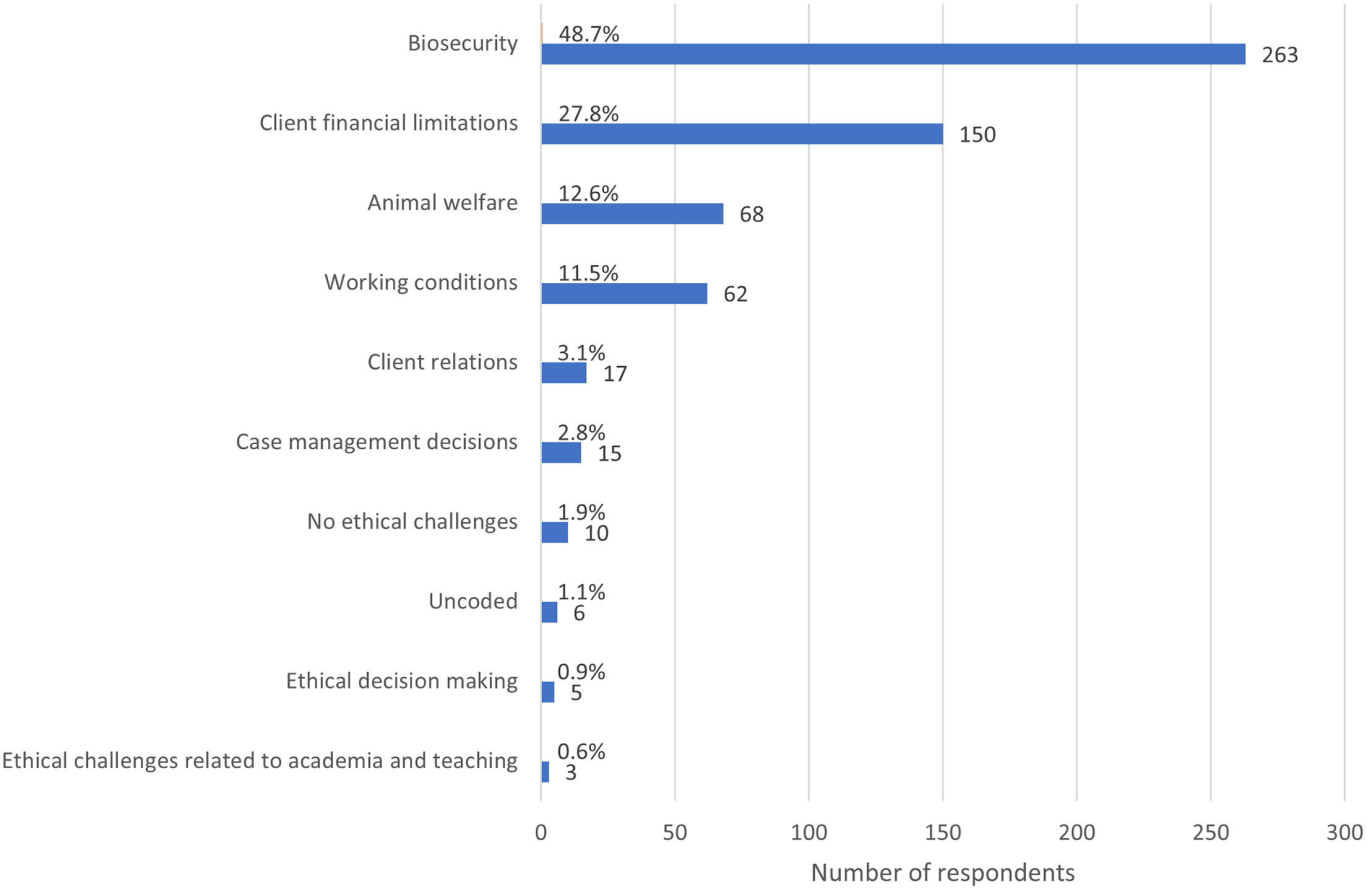
Bar chart of the frequency of major themes from respondent descriptions of the most common ethically challenging situations encountered since the advent of the COVID-19 global pandemic, based on the responses of 540 veterinarians, animal health technicians and veterinary nurses, surveyed between May and July in 2020. Note that a single response could be coded for multiple themes.

Overall, there were 17 major themes identified across responses to the three open-ended questions. When asked to describe the most common ECS since the advent of COVID-19, 540 respondents provided a comment (100%), providing 13829 words for analysis. The length of these comments ranged from 1 to 245 words. The most prominent themes were biosecurity (featuring in 48.7% or *n* = 263 responses), client financial limitations (27.8%, *n* = 150), animal welfare (12.6%, *n* = 68), working conditions (11.5%, *n* = 62) and client relations (3.1%, *n* = 17) (Figure 8).

**Figure 9:**
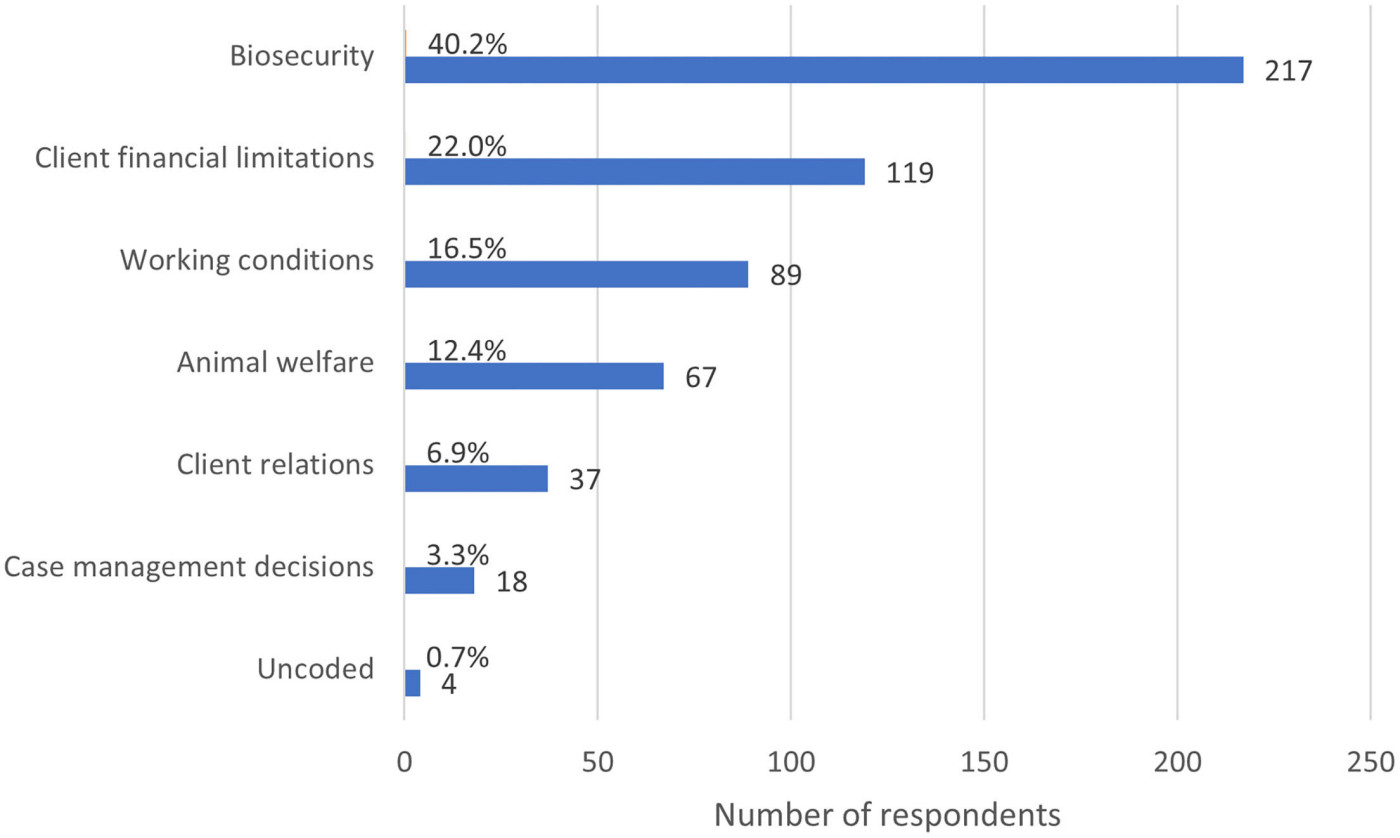
Bar chart of the frequency of major themes from respondent descriptions of the most stressful ethically challenging situations encountered since the advent of the COVID-19 global pandemic, based on the responses of 540 veterinarians, animal health technicians and veterinary nurses, surveyed between May and July in 2020. Note that a single response could be coded for multiple themes.

When asked to describe the most stressful ECS since the advent of COVID-19, all respondents provided a comment (*n* = 540), providing 10,234 words for analysis. The length of these comments ranged from 1 to 473 words. The most prominent themes were biosecurity (emerging in 40.2% of responses, *n* = 217), client financial limitations (22.0%, *n* = 119), working conditions (16.5%, *n* = 89), animal welfare (12.4%, *n* = 67), and client relations (6.9%, *n* = 37) (Figure 9). There was substantial overlap of themes between the first two questions, with 264 respondents reporting that the most common ECS they encountered since the advent of COVID-19 was also the most stressful.

Readers are referred to Supplementary Table 3 for excerpts from free-text responses illustrating themes and subthemes regarding the most common and most stressful ECS encountered by respondents since the advent of the COVID-19 pandemic.

**Figure 10:**
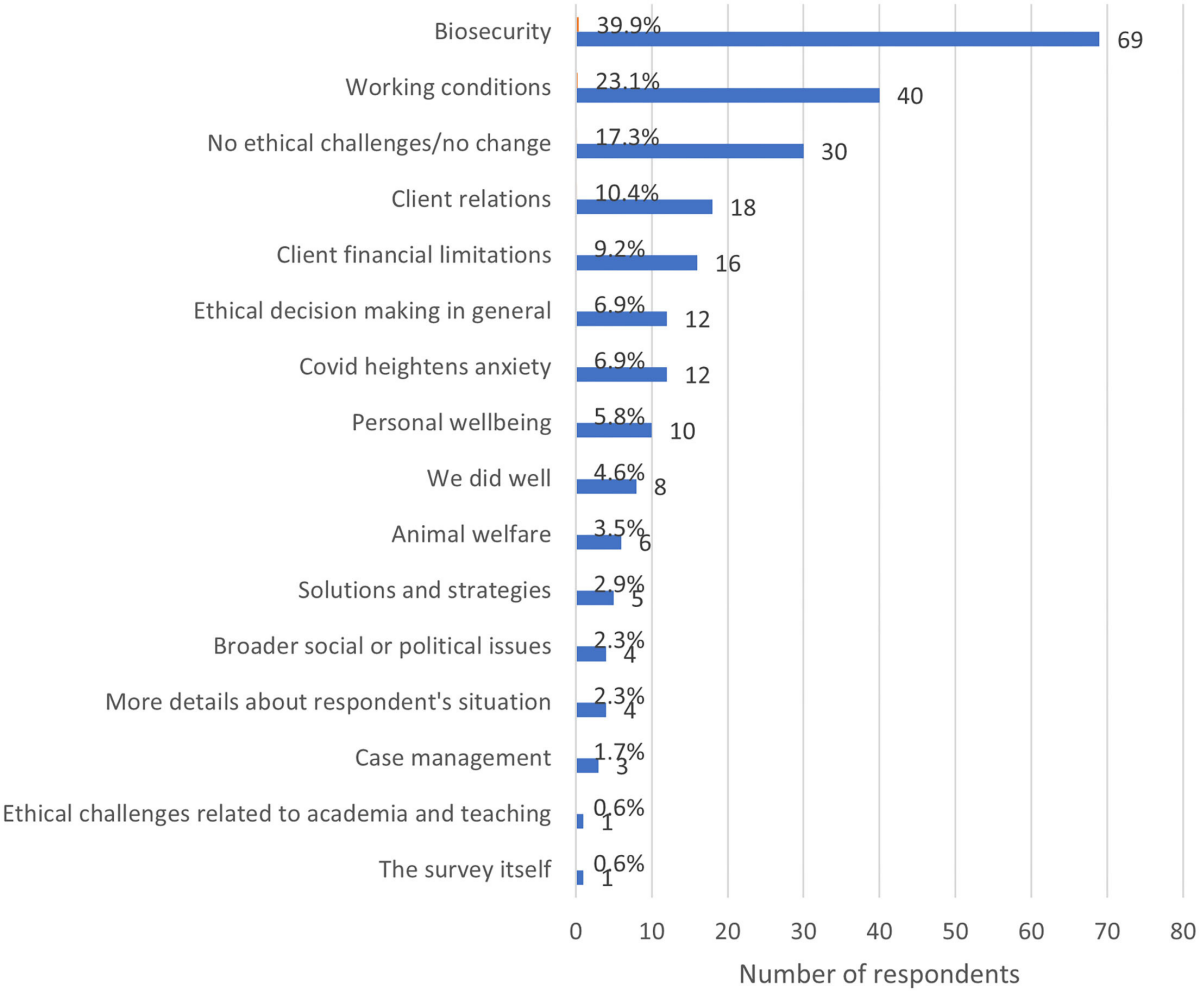
Frequency of major themes from respondent's additional comments regarding ethically challenging situations during the COVID-19 pandemic, based on the responses of 173 veterinarians, animal health technicians and veterinary nurses, surveyed between May and July in 2020. Note that a single response could be coded for multiple themes.

When asked if there was anything else they would like to add about their experience with ECS since the advent of COVID-19, 195 respondents (36.1% of the total sample) provided a comment. Of these, 22 wrote “no,” “none,” “n/a,” or “nil,” leaving 173 comments totaling 8038 words remaining for analysis. The length of these comments ranged from 2 to 298 words. Many respondents utilized this section to expand on themes they had already mentioned, particularly biosecurity (39.9%, *n* = 69), working conditions (23.1%, *n* = 40), the fact that they had not experienced ECS or that there was no change in the ECS they had experienced since the advent of the pandemic (17.3%, *n* = 30), client relations (10.4%, *n* = 18), and client financial limitations (9.2%, *n* = 16). The most prevalent new themes were COVID-19 heightening anxiety or stress in general (6.9%, *n* = 12), the challenge of maintaining personal wellbeing (5.8%, *n* = 10), and a sense that veterinary teams or the veterinary profession did well in a pandemic situation (4.6%, *n* = 8) (see Figure 10).

Readers are referred to Supplementary Table 4 for excerpts from free-text responses illustrating themes and subthemes from additional comments provided by respondents.

## Discussion

This is the largest global survey on ECS encountered by veterinary team members. The results of this study indicate that veterinary team members experienced increased frequency of ECS during a global pandemic. The median frequency of ECS encountered by veterinary team members increased from several times per month to several times per week with the advent of the COVID-19 global pandemic. The pre-pandemic frequency of ECS reported by veterinary team members is comparable with previous surveys on the frequency of ECS experienced by veterinarians. Pre-COVID-19 surveys suggested that veterinarians experience an ECS at least weekly, with 57% of UK veterinarians reporting 1-2 ethical dilemmas per week (range 0 to more than 10 times weekly) (*n* = 58) (4), 52% of US veterinarians experiencing an ECS at least weekly (*n* = 484) (3), and veterinarians, animal health technicians and veterinary nurses globally (*n* = 183) reporting a median of one ethical dilemma per week (22).

The increase in frequency of the ECS reported by respondents is likely due to a range of factors, including an increased frequency of established ECS such as client financial limitations, increased workload experienced by many veterinary teams, and the emergence of new or novel ECS associated with the COVID-19 pandemic itself.

We found that the COVID-19 pandemic was associated with both established and novel ECS in veterinary settings. The most common ECS, experienced by over two-thirds of respondents at least several times per week since the advent of the pandemic (64.4%, *n* = 348), was *challenging decisions about how to proceed when clients have limited finances*. This ECS was experienced as very or maximally stressful by 39.4% of respondents who encountered it (*n* = 195/495) of respondents, and was selected by 22.4% (*n* = 121) of respondents as the most recent type of ECS on which respondents chose to reflect. Additionally, client financial limitations emerged as a major theme in analysis of free-text responses, with respondents noting both an increase in client financial limitations reducing standard of care, and an increase in economic euthanasia.

Previous surveys have identified client financial limitations as common ECS encountered by veterinarians and veterinary team members. For example, veterinary anesthetists and technicians reported that animal care was impacted by financial constraints in 29% of ethically challenging cases (22). However, while identified as the most common ECS in some surveys of veterinarians, the same respondents reported client financial limitations as the least stressful ECS (2, 4). It has been speculated that this may be because financial limitations are accepted as common, that veterinarians may find a way of working within client cost constraints, or that cost constraints are seen as the client's responsibility (4). While this may have been the case prior to the pandemic, it is possible that the frequency and extent of client financial limitations exceeded the coping threshold of many veterinary team members. In the context of a global pandemic, the number of financially limited clients encountered by any one veterinary team member may become overwhelming, with fewer opportunities to work within cost constraints, recognition that clients who have lost jobs due to the pandemic are not responsible for their financial limitations, or a combination of these. In the US, 72% of small animal emergency hospitals reported that clients had more financial limitations than prior to the pandemic (23). It is likely that the long-term economic impacts of COVID-19, particularly large-scale unemployment (24), will decrease the accessibility of veterinary care for many people, compromising animal health and welfare. In addition, long-term economic consequences of the pandemic are likely to compromise regional, national and cross-border veterinary services (25). A survey of 565 British Veterinary Association Voice of the Veterinary Profession members found that up to 95% of respondents reported some level of concern about the potential impacts of a recession on the veterinary sector, with the most concern reported by government, charity and equine veterinarians (26).

The next most commonly encountered ECS was *conflict between personal well-being and professional role*, encountered by 64.3% (*n* = 347) of respondents at least several times per week. Similarly, almost half of respondents encountered *conflict between the well-being of family/household members and professional role* (46.3%, *n* = 250). Both of these ECS were experienced as very or maximally stressful by38.0% (194/510) and 33.6% (154/459) of respondents who encountered these, respectively. The impact of these challenges was underscored by emergence of biosecurity as the predominant theme in the thematic analysis. For example, an Australian veterinarian reported that it was stressful navigating the “high risk to myself for contacting [sic] disease or being a carrier and passing the disease on to my family” (respondent 466), while a veterinarian from China reported their most stressful ECS as “should I personally stop work to shield my vulnerable son but this will leave my colleagues and patients under more stress” (respondent 910). Veterinary team members had to struggle with the question of, as one US-based veterinarian put it, “what exposure limit is acceptable?” (respondent 780). As revealed in the thematic analysis, feeling torn between the risk of exposure to SARS-CoV-2 and the need to provide a service and/or support colleagues may have led to sickness presenteeism in some veterinary team members. While the impact of moral stress on the well-being of veterinary team members has been highlighted previously (2, 3, 5, 27), ECS arising due to the personal vulnerability of veterinary team members or their families to infectious disease have not been widely discussed.

Our thematic analysis revealed that ECS experienced during COVID-19 were often associated with uncertainty around biosecurity. It is possible that appropriate biosecurity guidelines, protocols and contingency plans may have reduced the conflict between personal well-being, and that of family or household members, and professional role, by ensuring that veterinary team members and organizations can operate with minimal risk to themselves, their colleagues and their families. These include strategies to discourage sickness presenteeism – which presents a risk to colleagues, clients and those in their networks – and encourage sickness absenteeism, such as paid pandemic leave for those required to self-isolate or undergo COVID-19 testing, and employment or contracting of trained staff to cover for those absences.

To this end, the pandemic exposed a lack of preparation among veterinary facilities. In their survey of small animal emergency hospitals in the US, Wayne and Rozanski reported that prior to the pandemic, fewer than half (44%) of hospitals had contingency plans for short-term disruptions such as snow days, while only 24% had disaster or business continuity plans. The remaining 32% had no plans for either short or long-term disruption (23). This is concerning, given the protracted disruption associated with the current pandemic, concern about subsequent “waves” of infection, and the possibility of intersections of COVID-19 with other disruptive events, including climate hazards and geopolitical issues (28).

Challenges arising from a conflict between personal well-being and professional role may arise in part due to uncertainty around the primary obligation of veterinary team members. Almost half (48.0%, *n* = 259) of respondents considered that ultimately, their primary obligation was to animal patients. However, the remainder of respondents were divided, revealing a lack of consensus among veterinary team members that may exacerbate moral conflict.

Tannenbaum described the veterinarian as the “servant of two masters” – human clients, on the one hand, and animal patients on the other (29). Rollin described the “fundamental question of veterinary ethics” as: “to whom does the veterinarian owe primary obligation – animal or owner?” (30). Rollin goes on to compare veterinarians who take the position that the animal is their primary obligation with pediatricians (30). But even prior to the pandemic, only 50% of US veterinarians reported that they prioritized patient interests, while only 20% reported that other practitioners prioritized patient interests (3). This survey did not reveal which interests were prioritized instead of patient interests. While it has been argued that veterinarians should be strong patient advocates, in acting for and advancing a case on behalf of patients and their interests (31), such a position may be challenging to maintain in a public health crisis, where the interests of animals and humans (including veterinarians and their families) are perceived to be in direct conflict.

Recognizing that most veterinarians are employed, in its Animal Welfare Strategy, the British Veterinary Association describes the veterinarian's trilemma as arising from duties to animals, clients and employers (32). It is interesting, therefore, that more respondents reported that they felt their primary obligation was to the community as a whole (13.0%, *n* = 70), other (12.6%, *n* = 68) or colleagues (10.2%, *n* = 55) compared to individual clients (8.2%, *n* = 43) and their employer (7.4%, *n* = 40).

There is a perception that in human healthcare, the primary obligation is – in theory – clearer. Oaths, such as the Hippocratic Oath, act as a moral compass in the face of ECS (33). According to the revised Declaration of Geneva, the health and well-being of the patient should be the first consideration (34). However, in most contexts, animals – unlike children – are considered the property of the owner, by law. Furthermore, the primary obligation of human healthcare professions shifts in the context of resource scarcity where the surge capacity of health-care systems is exceeded, and distributive justice must be explicitly considered. In the context of the COVID-19 pandemic, this was evidenced, for example, by the need for health care workers to determine how to triage human patients requiring mechanical ventilation in the face of a ventilator shortage (35). We asked respondents to report their primary obligation in a recent ECS. Whether veterinary team members' primary obligation changed in the context of a global pandemic, or even in the context of a specific ECS, could be examined in future studies.

*Conflicts between the interests of clients and the interests of their animals* emerged as both a common and stressful ECS encountered by veterinary team members, experienced by 59.6% of respondents (*n* = 322) at least weekly and experienced as very or maximally stressful by 50.2% (250/498) of respondents. This has been identified consistently in the veterinary literature as a common and stressful ECS. For example, in a survey of 889 North American veterinarians, 32% reported often having conflicts with pet owners about how to proceed with the care of their patients, while 53% reported having conflicts sometimes (5). In a survey of 484 small animal veterinarians in the US, 52% reported experiencing an ethical dilemma regarding the interests of clients and the interests of their patients at least weekly (3). Further investigation is required to determine the nature of these conflicts. For example, it is possible that some of these ECS involved situations in which the client's financial interests were in conflict with an animal's need for a certain standard of veterinary care, or veterinary care of any kind. They may also involve situations in which a client wishes to pursue treatment deemed by the veterinarian not to be in an animal's interests. It should be noted that in this context, the interests of animals are those perceived by the veterinary team member. It is possible that a client believes that they know the interests of their animal better than the veterinary team member, and, in some cases, they may be correct. Failure to recognize the perspective and relevant experience of another party may exacerbate conflict (36). This is a known gap, at least for veterinarians. In a survey of 889 veterinarians in North America, 71% reported that they had no training about resolving differences of opinion about what is best care for patients (5).

We found that 48.1% (*n* = 260) of respondents struggled with *challenging decisions about what counts as an essential veterinary service* at least several times per week, with 22.4% (*n* = 104/465) of respondents experiencing this ECS as very or maximally stressful. In the free-text comments, respondents reported struggling with determining what counted as an essential or emergent case – likely exacerbated by the absence of an end-date for pandemic-associated restrictions, and variation in official guidance and multiple waves of SARS-CoV-2 infection. This finding aligns with that of Wayne and Rozanski, who found variation in services that US-based veterinary hospitals would provide during the pandemic (23). Similarly, a round-table discussion on how the COVID-19 pandemic impacted the practice of avian and companion animal veterinary medicine revealed variation in both what was considered an essential service, and the safest way for veterinary hospitals to provide services (12). Respondents raised concerns about unintended consequences of uncertainty about what counts as an essential service, including delayed presentation of veterinary patients.

Part of the dilemma around the question “what counts as an essential service” is that the answer varies depending on the perspectives and time frame taken into account. As one UK-based veterinary nurse wrote, “Dental disease-not immediately life threatening but potentially may cause life altering issues if not treated” (respondent 430). It has been noted that some veterinary services, including preventative measures against diseases with a significant public health or economic impact such as rabies or tuberculosis, have been reduced or suppressed during lockdown (25, 37). This, combined with non-veterinary factors such as increased contact between wildlife and livestock, reduced population control, and longer on-farm stays of livestock, are likely to affect the distribution and incidence of transmissible animal diseases and zoonoses (25). Such programs may not be considered “essential” in that service reduction in the short-term may not compromise animal welfare or public health, but the reduction or absence of such services has the potential to cause significant harm over time.

Veterinary team members commonly reported having to make *challenging decisions about whether to perform non-contact veterinary visits*, with 46.3% (*n* = 250) encountering this ECS at least several times per week, with 17.9% (*n* = 80/447) finding this very or maximally stressful. While some respondents reported enjoying non-contact consultations, many reported struggling with communication, animal handling in the absence of the owner (particularly fearful aggressive animals), and non-contact euthanasia. This is not surprising. Much communication is non-verbal, and the inability to talk to an owner face-to-face may increase the risk of miscommunication or misunderstanding. As revealed by a number of respondents in free-text comments (see Supplementary Table 3), communication challenges were further exacerbated by PPE such as masks. As research reveals the impact of human-animal interactions on the welfare of animals (38), there has been increased awareness of the potential iatrogenic harms of veterinary care (39), and a profession-wide emphasis on minimizing fear, anxiety and stress in veterinary patients (40). The presence of a familiar person during a veterinary examination may provide a source of calm in what are otherwise likely to be perceived as threatening circumstances (41). In a small study of 32 owned dogs, dogs showed fewer indicators of fear when their owners were present (42). It is therefore unsurprising that a number of respondents raised concerns about the welfare of animals, the well-being of owners and the safety of veterinary team members when animals were examined away from the presence of their owners (see Supplementary Table 3).

In addition to impacts on animal welfare, non-contact euthanasia in particular may cause distress in clients. The veterinary euthanasia experience can alleviate or aggravate the grief of clients. A survey of 2354 pet owners in the UK conducted prior to the pandemic found that their experiences of administration practices (such as paperwork and payment), as well as emotional support at the time of the animal's euthanasia, were key influences on their satisfaction with the euthanasia experience (43). It is challenging for time-poor veterinary team members to provide streamlined administration practices and appropriate reassurance to clients. One Australian veterinarian expressed the ethical challenge thus: “in the case of very sick animals/emergencies/euthanasia's owners are distressed about not being able to be with their animal. Do you cave and let them be there knowing that if you get covid19 the entire clinic team and possibly other clients could get infected, or stick to the policy knowing you are causing emotional distress to the owner and animal?” (respondent 136).

In addition to the types of ECS that respondents could select from in the survey, a number of respondents specified “other” ECS, and indeed, these were experienced as very or maximally stressful by 42.9% (18/42) respondents who encountered them. This may reflect recall bias, where the ECS that comes to mind is the most salient to the respondent. Had the types of ECS specified as “other” been offered as choices that respondents could select from, it is possible that some would have been reported as very frequent. This information can be used to refine future studies on ECS, and incorporated into ethics teaching scenarios where possible.

Respondents used a range of strategies and resources to resolve ECS. Most respondents (66.3%, *n* = 358) found the resources and strategies they used somewhat helpful (35.9%, *n* = 194) or helpful (30.4%, *n* = 164), while 20.2% (*n* = 109) found them very or maximally helpful, and 6.3% (*n* = 34) didn't find them helpful at all. Additionally, 7.2% of respondents (*n* = 39) answered “not applicable” for this question.

Almost two thirds of veterinary team members (63.1%, *n* = 341) turned to discussion with colleagues in attempting to resolve ECS, an approach that has been documented previously. For example, a qualitative study of 7 small animal veterinarians in Australia found that discussing ECS and decisions with other veterinarians was a valued source of advice, facilitating benchmarking of their own experiences against those of others (44). However, this study also found that some veterinarians had experienced negative judgement of their ethical decisions by veterinarians and nurses, which may act as a barrier to discussion.

The next most frequently used resources were workplace policies (used by 32.2% of respondents, *n* = 174) and codes of professional conduct and/or veterinary oaths (used by 25.6% of respondents, *n* = 138). Codes of professional conduct and oaths are necessarily general, but can help guide those they apply to with regard to core professional values or primary obligation. Workplace policies are likely to be most useful with regard to expected ECS in known circumstances, for example client financial limitations (45) or managing requests for what veterinary team members deem to be futile care. Inflexible policies may also act as a barrier in some situations. While 32.2% of respondents used workplace policies to resolve an ECS, 23.1% (*n* = 125) identified workplace policies as a barrier. The quality and enforcement of policies may determine whether they help or hinder resolution of ethical challenges.

Ethical frameworks were knowingly used by only 15.2% (*n* = 82) respondents. This is consistent with a qualitative study which found that veterinarians tended to rely on “ethical intuition” rather than application of ethical frameworks to decide what to do/how to manage and ECS (44). A survey of veterinarians in the US (*n* = 484) found, alongside policies of state and national veterinary organizations, considerations of ethical theories were least commonly used in navigating ECS (3). In a study employing the Defining Issues Test to measure moral reasoning ability, practicing veterinarians in the UK performed similarly to members of the public, regardless of number of years in practice (1). In contrast, academic veterinarians had greater moral reasoning skills than both practicing veterinarians and the general public. The authors speculate that this may be a function of education, environmental factors (the normalization of exchange of ideas and opinions in academia), or a combination of factors. The use of ethical frameworks requires time, which may be scarce in practice settings, particularly during a global pandemic where many veterinary teams experienced an increased workload. In experimental settings, cognitive fatigue was shown to impact moral reasoning (46). Cognitive fatigue may be exacerbated by increased anxiety. A systematic review found that COVID-19 was associated with an increase in reported levels of psychological distress in the general population (47).

In addition, increased workload may lead to or exacerbate cognitive fatigue. Consecutive online surveys of 24-h small animal emergency veterinary hospitals in the US found that most reported caseload increases of at least 10%, with 44% reporting increases of at least 25% (48). Nonetheless, most hospitals had not made changes to operations or staff to accommodate these increases. Additionally, as our findings suggest, the impact of increased workload was exacerbated by staff shortages, including those associated with COVID-19 infection, potential exposure or other COVID-related absences (23, 48). In health care settings, inefficiency in workflow adaptation negatively impacts both quality of care and patient safety, while also eroding team cohesion and leading to moral stress of team members (49). This has the potential to further exacerbate cognitive fatigue.

Poor moral reasoning may lead to decision regret, rumination and moral stress, which negatively impact the well-being of veterinary team members. Importantly, insufficiently mature ethical reasoning or lack of ethical sensitivity may lead to negative animal welfare implications if veterinary team members cannot identify or effectively advocate for a course of action that is in an animal's interests (1, 50).

Almost all respondents (93.9%, *n* = 507) experienced at least one barrier to resolving an ECS to their satisfaction, with pressure from an employer or client [reported by 40.9% (*n* = 221) respondents] and client financial limitations (38.9%, *n* = 210) the most common. The pressure to generate income, a subtheme in the thematic analysis, may be a key reason for pressure from employers, while client financial limitations may be a key reason for pressure from clients. Both pressure to generate income and client financial limitations may be exacerbated with the ongoing pandemic. As these pressures are in direct conflict, if they occur concurrently they are likely to exacerbate stress on veterinary team members.

Though less common, *conflict between the interests of my employer and my own interests* [encountered by 35.0% (*n* = 189) of respondents at least several times per week], was experienced as very or maximally stressful by 42.5% (*n* = 178/419) of respondents who encountered it. *Conflict between the interests of my employees and my own interests* was experienced by 16.7% (*n* = 90) of respondents at least several times per week, and 22.9% (*n* = 59/258) found this very or maximally stressful. This is likely because of the power differential between employers and employees, as the stress experienced by employees may be heightened by fear of negative consequences such as change in working conditions or fear of losing their job. This is consistent with a non-peer-reviewed British Veterinary Association survey (*n* = 565) which found that 31% of veterinarians were quite concerned about job security (26). The thematic analysis revealed areas of conflict between personal interests and those of an employer, including disagreements around biosecurity. For example, a veterinarian in Germany reported feeling stress related to “working close together with colleagues that do not wear protection like face masks and therefore increase my risk to get infected (and this being announced as acceptable by my boss)” (respondent 79). Conflict may also arise around perceived pressure to generate income. For example, a US-based veterinarian reported “job threatened because I'm not producing enough revenue - told to charge more with no consideration to medical necessity” (respondent 89).

Interestingly, conflict between veterinary team members and their employers (as opposed to colleagues) has not emerged as an explicit theme in previous surveys of ethical challenges encountered by veterinary team members. The free-text responses suggest that factors contributing to the emergence of this conflict include conflict around pandemic measures, including biosecurity measures such as mask-wearing or determining what is an essential service. Other factors may include disagreements about workload management, perceived pressure to generate income, poor team morale do to concerns about job security (Supplementary Table 3), or general heightened anxiety (Supplementary Table 4).

Further studies are required to determine how the pandemic has exacerbated this conflict to a point of significance. Managing conflict between employers and veterinary team members has the potential to improve team morale and working conditions, as well as perceived job security.

Professional reassurance that their decision was the correct one was the leading form of assistance desired by respondents (46.7%, *n* = 252) when navigating an ECS. This may be one reason that so many veterinary team members turn to colleagues when faced with an ECS. Such reassurance may not be available in small teams or for professionals working in sole-charge settings. More than one quarter each of respondents desired additional help in mediating between conflicting points of view (26.1%, *n* = 141), and advice about potential alternative courses of action (25.7%, *n* = 139).

Despite these barriers, respondents reported overall a high degree of autonomy in making ethical decisions, with 70.4% (*n* = 380) reporting that they were free to make and act on ethical decisions always or most of the time. Autonomy is job resource associated with increased motivation and engagement, and consequently increased performance (51). Job demands may give rise to moral stress if veterinary team members feel constrained and unable to do what they believe is right. Because low decision latitude has been correlated with mental ill-health, increasing employee participation in decision making has been proposed as an organizational-level approach to improve psychological well-being in employees (52). According to Wallace, a feeling that one has a sense of discretion or control over these difficult situations may ameliorate moral stress (53). However, decision making autonomy is a double-edged sword. Job control may increase stress if, as we have seen in the context of a global pandemic, veterinary team members struggle with an overwhelming workload or demanding clients – situations which may be beyond their control (53). Perceived autonomy levels also differ among veterinary team members (54). The degree to which autonomy varied among different cohorts of respondents to the current survey will be discussed in a subsequent paper.

Our findings suggest that increased or better quality training of veterinary team members in navigating ECS may increase the strategies and resources available to them. Most respondents (54.3%, *n* = 293) had had some form of ethics training in obtaining their primary qualification, while 29.8% (*n* = 161) reported that they had none and 15.9% (*n* = 86) did not recall. This is consistent with a survey of 484 veterinarians in the US, which found that 51% of veterinarians reported having any ethics training during their veterinary degree (3). Of these, 39% agreed that it helped them navigate ECS, 38% were neutral, and 23% disagreed. In the same survey, 83.9% of respondents overall agreed with a need for veterinary school curricula to include training in ethical theories, and tools for coping with ECS. This compares favorably with an earlier study of 58 veterinarians in the UK, in which 78% reported inadequate training in ethics during their veterinary degree (4).

None of the above studies, including the current study, investigated the amount and quality of ethics training, nor its impact on the subsequent perception of frequency or stressfulness of ECS in veterinary team members. A survey of the American Veterinary Medical Association Council of Education (COE)-accredited institutions found that 18 of 30 offered a formal course in animal ethics (55). In a survey spanning 57 veterinary schools in 25 European countries, 72% of respondents reported that time spent teaching animal welfare ethics had increased or increased substantially (56). However, while the majority covered or exceeded requirements for animal welfare ethics (AWE) teaching, 37% of European veterinary education establishments only partially met or did not meet recommended Day-1 competencies for AWE. An online portal of shared resources in animal welfare and ethics was developed for veterinary students in Australia and New Zealand, but the extent to which its contents have been incorporated into curricula of regional veterinary schools is unknown (57). The effectiveness of ethics teaching may be impacted by the hidden curriculum – defined as unintentionally imparted and tacitly conveyed information about the culture of veterinary practice which may contradict overtly taught content (58). An example might be a curriculum that explicitly teaches shared decision-making, while being undermined by clinician teachers who are impatient or dismissive when talking to clients. When faced with this pedagogical mismatch, students are more likely to internalize values conveyed by the hidden curriculum (58, 59).

Most (51.7%, *n* = 279) of the current respondents reported undertaking some form of post-qualification ethics training, with continuing professional development the most common format (33.0%, *n* = 178). This suggests that there may be opportunities for veterinary team members who received little or no training in ethics in their formal curriculum to redress this deficiency by providing focused CPD. Furthermore, with 48.3% (*n* = 261) of respondents not having undertaken any post-qualification training or education in ethics, there is scope to expand this. We plan to explore whether post-qualification ethics training better equips veterinary team members in navigating ECS in a subsequent report on this study.

In the current study, very few respondents (4.6%, *n* = 25) reported an ideal resolution to an ECS, suggesting that such an ideal – while possible – is relatively uncommon. Crane and colleagues found that veterinarians who encountered morally significant stressors on their work tended to experience greater negative emotions if they were high in trait perfectionism (2). According to the authors of that study, veterinarians with perfectionistic or rigid standards are more likely to consider ECS as being “black and white” or clear cut, and are more vulnerable to reduced well-being due to ECS. In contrast, veterinarians lower in trait perfectionism were less likely to see only one resolution as right, and more likely to see a number of potential acceptable resolutions. If a less optimal resolution is achieved, these veterinarians were less likely to find the resolution unacceptable, and less likely to experience moral stress. When it comes to ECS, the authors concluded that “the goal of perfection throughout one's working life is for many veterinary practitioners likely to be impossible and largely impractical” (2).

Despite concerns raised in the current study, 82.0% (*n* = 443) veterinary team members overall reported that they were confident or reasonably confident that they could manage ECS in their workplace.

It is argued that moral distress or moral injury arising from ECS are indicators of problems with healthcare systems rather than individual team members working within them (60). In veterinary contexts, pandemic associated ECS (for example, conflict between personal well-being and professional role) must be addressed beyond the level of the individual. Given the likelihood of transboundary threats such as climate change, large-scale immigration, water and food shortages and cyber terrorism in the future (6), as well as local crises, it is important to understand and learn from the ECS encountered in the COVID-19 pandemic, and develop appropriate resources to equip veterinary team members to successfully manage these challenges.

### What Can Veterinary Teams Do to Prepare for Ethical Challenges?

Client financial limitations, already the most common ECS faced by veterinary team members, occur commonly in veterinary settings, but are exacerbated in the context of a pandemic. We therefore recommend that veterinary team members, veterinary facilities, professional organizations, Governments and non-government organizations prepare to accommodate clients with financial limitations, and take steps to increase access to veterinary care. This requires a multifactorial approach, combining strategies from animal health insurance and third-party credit to low-cost clinics, access to emergency funds for veterinary care and preventative programs, including disease surveillance, and continuing education of policy makers and the public about the importance of animal health and welfare. The Access to Veterinary Care Coalition have already outlined a number of potential strategies to expand access to veterinary care for companion animals (61). These should be explored as a matter of urgency, along with strategies to ensure continuity of veterinary care for large animals, zoo and wildlife animals, laboratory animals and other animals dependent on humans.

In the context of the pandemic, veterinary team members were faced with the dilemma of balancing their personal well-being – and the well-being of their family or household members – against their professional obligations. This is not a new dilemma. Veterinary team members are at potential risk of exposure to zoonoses. However, the focus of training is typically prevention of animal to human disease transmission. In the authors' experience, the COVID-19 pandemic is the first time there has been widespread awareness of the risks presented to veterinary team members from each other and clients. The dilemma of whether to prioritize personal safety over professional role can never be entirely eliminated. However, evidence-based, appropriately implemented biosecurity protocols can reduce risks associated with providing veterinary services. Such protocols must be clear, able to be adopted by all veterinary team members, and incorporated into training programs and continuing professional development. Additionally, veterinary clientele need to be informed about such protocols and educated regarding their rationale.

To be effective, biosecurity protocols should incorporate strategies to reduce sickness presenteeism. This will require significant cultural change. A global survey on sickness presenteeism comparing the self-reported behavior of health care workers and non-healthcare workers with influenza like illness found that the majority of both groups would continue to work, despite health care workers knowing the risks of transmitting influenza-like illness to vulnerable patients (62). Possible reasons for sickness presenteeism included understaffing, a sense of obligation to colleagues, and economic reasons such as lack of sick leave – all of which exist in veterinary settings. While some veterinary team members may see it as a moral obligation not to let their team members down through their absence due to mild signs of illness, the COVID-19 pandemic has highlighted the potential negative consequences of sickness presenteeism, including exposure of employers to complaints and liability for failing to prevent exposure of employees to infection (63). The taking of sick leave to undergo testing or isolation for COVID-19 or indeed any other infectious disease must be accepted as an important means of protecting staff, colleagues and clients, and in some cases the viability of a veterinary service itself. However, to facilitate this cultural shift and avoid pressure on those with symptoms, veterinary facilities must develop contingency plans for staff absence due to illness. Where possible, paid sick leave should be made available to remove economic barriers to sickness absenteeism. As paid sick leave will not address sickness presenteeism arising from a sense of obligation to the veterinary team, it is important that practices proactively develop contingencies for staff taking leave, including employing additional team members, which may also assist with the workload, training staff to undertake a broader range of duties within their scope of practice, or contacting locum agencies or developing relationships with trained casual/ temporary staff to be on standby (64).

Conflicts between the interests of animals and their owners were commonly reported by veterinary team members in this and previous surveys. Further information is required to understand the nature of such conflicts, for example, whether these emerge from different beliefs about the moral status of animals, differences of opinion between owners and veterinary team members regarding the level of suffering an intervention or lack of intervention may cause, conflicts resulting from insufficient information or evidence, differences in values between veterinary team members and clients and so forth. Understanding the bases of these conflicts is an critical in communicating about and potentially resolving them (65). For example, a veterinary team member may perceive a client's request to euthanase an animal with a treatable condition as a conflict between the interests of the owner and the animal. But that request may stem from a genuine concern, on the part of the owner, about the quality of life of the animal once treatment is commenced. In this case, understanding the basis of the client's objections to treatment may be the first step in reassuring the client that the treatment would in fact improve the animal's quality of life. Meaningful communication requires time to explore values and to find common ground between veterinary team members and clients. Communication skills can be taught and, like all skills, be constantly honed and developed by veterinary team members (36).

Many respondents reported that they referred to their professional oath or code of conduct in resolving ECS. We recommend that professional organizations and registration bodies consult with their stakeholders about how these documents help or hinder resolution of ECS in the context of the COVID-19 pandemic. For example, it may be that in some cases, these documents provide clarity or confusion around the primary obligation of veterinary team members, the types of services considered essential or the role of veterinary team members in an emergency. This information should be compiled and used to refine oaths and codes, to ensure that these resources are as helpful as possible for those navigating ECS. Individual veterinary team members may wish to review their oath and code of professional conduct in the light of the challenges they faced, and provide feedback proactively to their respective regulators and boards.

Pressure from an employer or client was viewed as a major barrier to resolution of ECS by respondents in this survey. To overcome pressure from employers, those studying moral injury in the human healthcare field recommend bringing the “employers” (administrators) and “employees” (clinicians) together, to understand each other's respective roles and responsibilities. It has even been recommended that individuals from each of these groups “shadow” their counterparts (60). The rationale is to appreciate the unique stressors and challenges faced by each group, and to establish common ground from which compromises may be found. Such an approach could be encouraged and supported by professional organizations.

While most respondents had had some form of ethics training, few employed ethical frameworks to aid in decision making. There is scope for veterinary educators to develop curricula and continuing professional development allowing attendees to work through ECS that may be encountered in the context of a pandemic, such as those outlined in this paper, in a psychological safe environment, without time pressure.

Veterinary teams can establish structures to provide advice about alternative courses of action, help in mediating conflicting perspectives and (where appropriate) professional assurance that the best, least worst or right course of action was taken. Discussion of active ECS with an ethics committee may address these needs, though there are practical and resource constraints to consider (66, 67). However, it may be possible to meet at least the first need in ethics rounds, or indeed in morbidity and mortality (M&M) rounds (68, 69). If a decision was inappropriate, questionable or incorrect, sensitive, non-judgmental debriefing in M&M or ethics rounds may be helpful. There may be important contextual and practical reasons why a particular decision was made. Exploration of these factors may be used to refine future decision making, including policies and protocols, as occurs in root cause analysis of medical errors (70, 71). All veterinary team members have a role in steering away from a culture of blame – which acts as a barrier to reporting and appropriate debriefing, and promoting a culture of learning from errors (72). Ethics rounds have been shown to improve moral reasoning and may improve ethical awareness or ethical sensitivity among medical students (73, 74), but their impact in veterinary settings remains to be explored.

We believe that it is important for veterinary team members to appreciate that the primary resource utilized in navigating ECS was discussion with colleagues, relied upon by almost two-thirds of respondents. Discussion of ECS with colleagues may be a means of identifying all stakeholders, identifying alternative approaches or options, or simply as a means of being reassured that one has not overlooked an obvious stakeholder or option, and made the best possible decision in the circumstances. It may also be a means of learning that a different approach might have been better, and could be a vital learning opportunity. However, as has been previously recognized (44), negative judgement from colleagues may act as a barrier to discussion. Training in communication, including reflective listening, provision of constructive feedback and conflict management may facilitate improved discussion between colleagues. In addition to training, veterinary team members need time and space to talk to colleagues. Where physical distancing precludes face-to-face discussion, it may be possible to set up online one-on-one and team meetings for this purpose.

The COVID-19 pandemic, like previous pandemics, has highlighted the problematic nature of human-animal interactions, with human behaviors such as incursion into wildlife habitat, habitat destruction, unnatural human-animal contact, the consumption of wildlife, overcrowding of animals, live animal markets and transport of animals being identified as risk factors for the spread of zoonotic disease (75, 76). However, our findings suggest that, during a time of crisis, veterinary team members are preoccupied with proximate concerns and may not have time to address these “wicked” problems. The need to plan and prepare veterinary services in advance of crises such as pandemics, and to provide coordinated, appropriate management in response to such crises has been discussed previously (77, 78), but such a need competes with economic reality and inertia. Given the impact of the COVID-19 pandemic on the veterinary team members, the animals and communities we serve, veterinary professionals should take steps to address the underlying causes, at the level of facilities, communities and organizations.

### Limitations

A major limitation of this study is its inability to characterize the source population from which respondents were sampled. The exact populations of veterinarians, animal health technicians and veterinary nurses globally are unknown, so a response rate could not be calculated.

Where possible, we asked veterinary, nursing and animal health technicians organizations to distribute the link to our survey to their members via electronic mailing lists (see Supplementary Table 2). This non-random, *ad-hoc* sampling method may have biased selection to respondents who were more interested in ethics or ECS, or biased selection toward certain cohorts. For example, most of the organizations that agreed to distribute the link were veterinary boards or organizations, which may have biased selection toward veterinarians rather than veterinary nurses and animal health technicians. The offer of an incentive may have increased participation.

Unrestricted, open surveys introduce the risk that respondents may not be who they say they are, that respondents may complete the survey multiple times to create a “ballot box stuffing” effect, or that web robots may be used to generate spam data (17). In this case, every response was carefully reviewed, and all responses contained unique, detailed information indicating that, on balance, the data are likely to be legitimate. A disadvantage of anonymity is that we could not provide support to individuals who expressed strong negativity, other than providing very general information about support services at the conclusion of the survey (79).

Questionnaire design may have influenced respondents. For example, a respondent may not previously have considered a potential ECS before reading this option in this question. However, the first two questions in the survey asked respondents to describe the most common and most frequent ECS they encountered in their own words before proceeding to the next section. This encouraged respondents to consider the ECS they had encountered before suggesting any particular types of ECS.

The open-ended questions provided space for participants to describe situations that they encountered, but the anonymity of responses meant that further clarification was not possible. Thus, it is possible we might have misunderstood certain responses, leading to inappropriate categorization in the thematic analysis.

The length of the survey may have discouraged potential respondents. Indeed, many who did take the time to complete the survey indicated that they were time-poor and overworked, and the pandemic has been associated with increased rates of burnout among veterinary team members in some contexts (48). A shorter survey may have captured a greater breadth of responses.

Finally, this survey can only provide a cross-sectional snapshot of ECS faced by veterinary team members during a brief time period (May to July 2020). At the time of publication, many countries and regions are experiencing subsequent waves of the pandemic. The COVID-19 pandemic has been described as a “creeping crisis,” with undefined end-points, no clear path to exit from restrictions, and potential to “change shape along the way” (6), causing challenges that are much harder to manage than those generated by acute crises that are more sharply delineated in time. It is likely, therefore, that veterinary team members may experience different and perhaps even totally unique ECS, associated with varying degrees of stress, at different time-points in the pandemic.

## Data Availability Statement

The datasets generated for this article are not readily available because we have approval to disseminate aggregated data, but not individual data. Requests to access the datasets should be directed to anne.quain@sydney.edu.au.

## Ethics Statement

The studies involving human participants were reviewed and approved by The University of Sydney Human Research Ethics Committee approval number 2020/291. The patients/participants provided their written informed consent to participate in this study.

## Author Contributions

AQ: literature review, study design, survey building and piloting, ethics application, data analysis, writing, editing, and submission. SM: study design, survey refinement, ethics application, data analysis, editing, and supervision. PM: study design, survey refinement, ethics application, editing, and supervision. MW: data analysis, editing, and supervision. All authors contributed to the article and approved the submitted version.

## Conflict of Interest

The authors declare that the research was conducted in the absence of any commercial or financial relationships that could be construed as a potential conflict of interest.
