# Supplementary material for: Frequency, Stressfulness and Type of Ethically Challenging Situations Encountered by Veterinary Team Members During the COVID-19 Pandemic
Source: Front Vet Sci. 2021 Apr 12;8:647108. doi: 10.3389/fvets.2021.647108 (PMC8071942; doi:10.3389/fvets.2021.647108)
Supplement: Supplementary file 1 [file Data_Sheet_1.docx]

Supplementary Material

**Supplementary Table 1.** Survey on ethically challenging situations encountered by veterinarians, animal health technicians and veterinary nurses in the COVID-19 era available online from May to July 2020.

| **Please answer the following questions about ethically challenging situations. For the purposes of this survey, an ethically challenging situation is defined as a situation where we are required to manage competing choices, or where there may be conflict between the interests of different stakeholders or parties who may be impacted by a decision.** |
| --- |
| Prior to the advent of COVID-19, how often would you experience an ethically challenging situation? (Please choose one option)   - Less than once per month - Several times per month - Several times per week - Daily - Several times per day - Never   Since the advent of COVID-19, how often would you experience an ethically challenging situation as veterinary team member? (Please choose one option)   - Less than once per month - Several times per month - Several times per week - Daily - Several times per day - Never   Since the advent of COVID-19, describe the most COMMON ethically challenging situation you have encountered as a veterinary team member? (This does not have to be specific to COVID-19. Please be careful not to include potential identifying information such as the names of individuals or workplaces in your response).  Since the advent of COVID-19, describe the most STRESSFUL ethically challenging situation you have encountered as a veterinary team member? (If the response is the same as above, enter “same”. (This does not have to be specific to COVID-19. Please be careful not to include potential identifying information such as the names of individuals or workplaces in your response). |
| The following is a list of situations where decision may be ethically challenging. How often have you faced these? |
| Challenging decisions about distribution of personal protective equipment  Challenging decisions about distribution of other equipment such as ventilators  Challenging decisions due to a lack of resources  Challenging decisions about how to proceed when clients have limited finances  Conflict between the interests of clients and the interests of their animals  Conflict between the interests of colleagues and my own interests  Conflict between the interests of my employer and my own interests  Conflict between the interests of my employees and my own interests  Challenging decisions about what counts as an essential veterinary service  Challenging decisions about whether to perform non-contact veterinary visits  Conflict between personal wellbeing and professional role  Conflict between the wellbeing of family/household members and professional role  Challenging decisions about how to manage captive wildlife  Challenging decisions about how to manage free-ranging wildlife  Challenging decisions about how to manage feral or declared pest animals  Challenging decisions about management of laboratory animals  Challenging decisions regarding human resources during economic downturn  Challenging decisions about whether to use skills for animal health or human wellbeing  Challenging decisions about the provision of practical experience or training to veterinary/animal health technician/nursing students  Other (Please describe. Please be careful not to include potential identifying information such as the names of individuals or workplaces in your response).  For each item listed above:   - Monthly - Several times per month - Several times per week - Daily - Several times per day - Never   How stressful did you find each of the following ethically challenging situations? (*i.e. those listed 1-20 above*) (select “not applicable” if you have not encountered this ethically challenging situation).   - Not stressful at all - A little stressful - Moderately stressful - Very stressful - Maximally stressful - Not applicable |
| In answering the following questions, please consider the most recent situation where you felt significant difficulty deciding upon the ethically right thing to do. Please choose a situation that has run its course. The example can come from any aspect of patient care or any other kind of situation in your workplace. Please answer the following questions in relation to that situation.  What type of situation was this (please choose one option):  Challenging decisions about distribution of personal protective equipment  Challenging decisions about distribution of other equipment such as ventilators  Challenging decisions due to a lack of resources  Challenging decisions about how to proceed when clients have limited finances  Conflict between the interests of clients and the interests of their animals  Conflict between the interests of colleagues and my own interests  Conflict between the interests of my employer and my own interests  Conflict between the interests of my employees and my own interests  Challenging decisions about what counts as an essential veterinary service  Challenging decisions about whether to perform non-contact veterinary visits  Conflict between personal wellbeing and professional role  Conflict between the wellbeing of family/household members and professional role  Challenging decisions about how to manage captive wildlife  Challenging decisions about how to manage free-ranging wildlife  Challenging decisions about how to manage feral or declared pest animals  Challenging decisions about management of laboratory animals  Challenging decisions regarding human resources during economic downturn  Challenging decisions about whether to use skills for animal health or human wellbeing  Challenging decisions about the provision of practical experience or training to veterinary/animal health technician/nursing students  Other (Please describe. Please be careful not to include potential identifying information such as the names of individuals or workplaces in your response).  I considered that ultimately, my primary obligation was to: (please choose one option)   - Individual animal patients - Individual clients - My employer - My colleagues - Conservation of species - The Government - The community as a whole - Other (please specify)   How stressful did you find this situation? (please choose one option)   - Not stressful at all - A little stressful - Moderately stressful - Very stressful - Maximally stressful - Not applicable   Which of the following did you employ when faced with this ethically challenging situation? (select all that apply)   - Use of an ethical decision-making framework (for example, an ethical matrix, a cost:benefit analysis, a utilitarian framework such as the 3Rs) - Reference to a code of professional conduct and/or veterinary oath - Workplace policies - Consultation with an ethics committee - Discussion with colleagues - Referring the decision to a colleague - Refer to the published literature specifically about ethics - Consultation with a health care professional (e.g. psychologist, counsellor) - Consultation with a community leader or priest - Discussion with a spouse or partner - None of the above - Other (please specify)   How helpful were these strategies in enabling you to resolve this issue? (please choose one option)   - Not helpful at all - Somewhat helpful - Helpful - Very helpful - Maximally helpful - Not applicable   How would you rate the acceptability of the eventual outcome? (please choose one option)   - Unacceptable - Uncertain - Acceptable, could be improved - Good - Ideal   What were the barriers to resolving ethical issues to your satisfaction? (Please select all that apply)   - Lack of time - Financial limitations - Difficulty in communicating with clients - Difficulty in communicating with colleagues - Pressure from an employer or client - Differences in values between stakeholders - Conflicts of interest (mine) - Conflicts of interest (another stakeholder) - Workplace policies - Concerns about liability - I am not aware of any barriers to resolving ethical issues to my satisfaction - Other (please specify)   In reflecting back on this case, which of the following types of assistance or resources would you have found useful? (please select all that apply)   - Additional help in clarifying the ethical issues for yourself, your patient or your colleagues - Additional help in obtaining more complete information from what you had available to you - Additional help in mediating conflict among different points of view - Alternative suggestions for ethically appropriate courses of action - Professional reassurance that your decision was the correct one - None of the above - Other (please specify) |
| We are now going to ask you some demographic questions, as well as some questions about your training, experience and role. |
| I am a: (please choose one option)   - Veterinarian - Animal health technician - Veterinary nurse - Other animal health professional   In which country do you work?   - Drop down menu listing all countries   In which year did you graduate?   - Drop down menu listing years 1940-2020   In which year were you born?   - Drop down menu listing years 1920-2003   What is your gender?   - Female - Male - Other   What constitutes the majority of your workload? (please choose one option)   - Companion animal practice clinical - Mixed animal practice clinical - Exotic/unusual pet practice clinical - Zoo and/or wildlife practice clinical - Equine practice clinical - Practice management - Academia/teaching - Scientific research/laboratory animals - Government - Non-Government organisation - Industry (e.g. pharmaceutical companies, food companies) - No longer working as a veterinarian - Other (please describe what constitutes the majority of your workload in words)   How many hours per week do you work in your role as a veterinarian, animal health technician or veterinary nurse? (select one option)   - 0-10 - 11-20 - 21-30 - 31-40 - 41-50 - 50+   In obtaining your qualification for your current role, where you taught specifically about ethics? (select one option)   - Yes - No - Don’t recall   Have you undertaken any of the following since qualifying as a veterinarian/animal health technician or veterinary nurse? (please select all that apply)   - University coursework (diploma/degree) in ethics or bioethics - Continuing professional development in ethics - Sat on an institutional ethics committee - Other (please specify)   How confident do you feel in dealing with ethically challenging situations in your workplace? (please choose one option)   - Not confident at all - Underconfident - Confident enough that I can get by - Reasonably confident - Couldn’t be more confident   In my workplace, I feel that I am free to make and act on ethical decisions: (select the option that applies to you most of the time)   - Never - Rarely - Sometimes - Most of the time - Always   Is there anything else you would like to add about your experience with ethically challenging situations since the advent of COVID-19? (Please be careful not to include potential identifying information such as the names of individuals or workplaces in your response). |

**Supplementary Table 2.** Professional organisations, professional bodies and special interest groups who shared the link to the survey on ethically challenging situations encountered by veterinarians, animal health technicians and veterinary nurses in the COVID-19 era available online from May to July 2020.

| **Parties sharing the research invitation on behalf of the study team** | **Mode of recruitment** | **Relevance to target population** | **Professional background targeted** | **Country targeted** |
| --- | --- | --- | --- | --- |
| World Veterinary Association | Facebook and Twitter accounts | Professional association | Veterinarians | International |
| Veterinary Surgeon’s Board, Western Australia | Email newsletter | Professional Body | Veterinarians | Australia |
| Veterinary Board of Tasmania | Email | Professional body | Veterinarians | Australia |
| One Welfare World | Twitter account | Professional interest group | Veterinarians, animal health technicians, veterinary nurses | International |
| Cat Protection Society NSW | Facebook page | Animal welfare organisation | Veterinarians, veterinary nurses | Australia |
| British Veterinary Association | Email | Professional association | Veterinarians, veterinary nurses | UK |
| Commonwealth Veterinary Association | Email | Professional association | Veterinarians | International |
| Veterinary Public Health Institute, University of Bern, Switzerland | Email | University | Veterinarians | Switzerland |
| Federation of Asian Veterinary Associations | Website | Professional association | Veterinarians | Asia |
| European College of Animal Welfare and Behaviour Medicine | Facebook | Professional association | Veterinarians | Europe |
| Veterinary Practitioner’s Board of NSW | Newsletter | Professional body | Veterinarians | Australia |
| COVID-19 Daily Digest for Animal Shelters | Newsletter | Multiple animal welfare organisations | Veterinarians, animal health technicians, veterinary nurses | North America |
| Veterinary Nurses Council of Australia | Facebook | Professional association | Veterinary nurses, animal health technicians | Australia |
| Australian and New Zealand College of Veterinary Scientists (ANZCVS) | College newsletter; animal welfare chapter newsletter | Professional association | Veterinarians | International |
| Sydney School of Veterinary Science | Facebook; Twitter; staff newsletter | University | Veterinarians, animal health technicians, veterinary nurses | International |
| Australian Veterinary Association | Facebook; newsletter | Professional association | Veterinarians | Australia |
| Human Society Veterinary Medical Association | Facebook | Professional association | Veterinarians; animal health technicians; veterinary nurses | International |
| Getting 2 Zero | Facebook; newsletter | Animal welfare organisation | Veterinarians; animal health technicians; veterinary nurses | International |
| Vets Beyond Borders | Facebook; newsletter | Animal welfare organisation | Veterinarians; animal health technicians; veterinary nurses | International |
| Centre for Veterinary Education | Facebook; newsletter | Continuing Education provider | Veterinarians | International |
| Society for Veterinary Medical Ethics | Electronic mailing list | Professional association | Veterinarians; animal health technicians; veterinary nurses | International |

**Supplementary Table 3.** Key themes identified in thematic analysis of free-text responses to online survey questions regarding the most common and most stressful ethically challenging situations (ECS) encountered by veterinarians, animal health technicians and veterinary nurses since the advent of the COVID-19 global pandemic (n=540).

| **Theme** | **Sub-theme** | **Examples** |
| --- | --- | --- |
| Biosecurity | Non-contact consultations (in general) | *“The balance between upholding social distancing in the clinic and being sympathetic to the emotional needs of clients who want to stay with their pets”* (531, veterinarian, Australia).  *“Delivering bad news in a non-private situation (in the carpark, usually with other owners nearby)”* (317, veterinarian, Australia).  *“Not allowing animal caretakers and managers into the zoo hospital during procedures and having to manage and mitigate the increased communication and education between veterinary team members and animal caretakers that is necessary as a result”* (504, veterinarian, USA).  *“Keeping owners out of the consult has been wonderful, better than before. Owners more focussed and attentive to their pets”* (342, veterinarian, Australia). |
|  | Non-contact consultations (euthanasia) | *“Not allowing people to visit or accompany their dying pets into emergency”* (361, veterinary nurse, Australia).  *“Limiting number of clients present for euthanasia and not being able to provide physical reassurance or hugs”* (318, veterinarian, Australia).  *“I was called out to an after-hours call on a collapsed dog. The dog died just minutes before arrival at the clinic. We tried to revive it to no avail. It was the middle of the night, freezing cold and pouring with rain and we were about to wrap the body up and take it away and the owners wanted some time with it to say goodbye. We couldn't let them inside so they had to say goodbye outside where they and the dog got absolutely saturated, cold, and couldn't even see the dog properly”* (115, veterinary nurse, Australia).  *“in the case of very sick animals/emergencies/euthanasia's owners are distressed about not being able to be with their animal. Do you cave and let them be there knowing that if you get covid19 the entire clinic team and possibly other clients could get infected, or stick to the policy knowing you are causing emotional distress to the owner and animal?”* (136, veterinarian, Australia).  *“Making the decision to allow owners into the clinic for euthanasia of their pet. I was happy to do this, but at the same time thought of a friend of mine who died and I was unable to attend his funeral”* (126, veterinarian, Australia). |
|  | Impact of social distancing on animal welfare and safety of the veterinary team. | *“Examining dogs (and cats) without the owner present has more frequently resulted in the dog being very anxious (often with risk of aggression towards myself and the vet nurse assisting me)”* (332, veterinarian, Australia).  *“Trying to get pets to come inside with me while wearing a mask, gloves and gown (which is super scary to them) while the owner stays in the car. They usually try to pull back towards their owner or won't move at all. (mostly only dogs since cats are in carriers) It makes it scary that I might lose them especially since we are on a busy road and near two busy train routes. We try to bring out two leashes to control them but they are slip leads and tend to choke them if they pull away hard”* (47, veterinarian, Australia).  *“The most stressful experiences involve traversing the need for social distance between humans with many of the animals' needs for their people to be present to comfort them during medical evaluation. I run a low-stress/fear-free hospital, so I see more than my share of anxious or aggressive patients. Treating them fairly is especially tricky right now”* (187, veterinarian, USA). |
|  | Conflict between provision of veterinary services and public health | *“Providing animal care but in so doing risking the health of my team, the clients and myself, and by staying open risking the spread of the pandemic”* (50, veterinarian, Australia).  *“I sit on a board for a veterinary charity and need to consider the risks and welfare of the people and animals who are involved in the charity, including volunteers/staff and also beneficiaries and their pets. This includes deciding to suspend services or continue to run services (both carry risks and benefits)”* (407, veterinarian, Australia).  *“If we were doing the right thing by humanity continuing performing routine procedures using up precious PPE and exposing ourselves to close interactions with team members and the public”* (465, veterinarian, Australia).  *“Choosing between animal health and welfare and the health and wellbeing of staff”* (524, veterinarian, USA). |
|  | Conflict between personal role and personal wellbeing or family wellbeing | *“high risk to myself for contacting [sic] disease or being a carrier and passing the disease on to my family”* (466, veterinarian, Australia).  *“Should I personally stop work to shield my vulnerable son but this will leave my colleagues and patients under more stress”* (910, veterinarian, China)*.*  *“What exposure limit is acceptable?”* (respondent 780). |
|  | What counts as an essential service | *“Having to decide if a case is considered "essential" or "non-essential". Sometimes it may seem like something that can be delayed, but it depends on the owner's ability to administer proper care. On a daily basis, we need weigh the potential for the condition to get worse against the public health and safety risk of having the client bring the pet in”* (83, veterinarian, Singapore).  *“Decisions on delaying procedures eg neutering/vaccination that aren't considered essential now but overall I feel are essential for animal welfare”* (108, veterinarian, United Kingdom).  *“My work focuses on equine (Thoroughbred) reproduction. The ethical challenge was whether this work could be classified as 'essential' during the lockdown period. Guidance from [identifier removed] was inadequate and confusing”* (213, veterinarian, Republic of Ireland).  *“Trying to decide what is life threatening and what isn’t. Dental disease-not immediately life threatening but potentially may cause life altering issues if not treated”* (430, veterinary nurse, United Kingdom). |
|  | Conflict about essential service | *“Clients demanding to be seen as an emergency when it is patently not an emergency and already full to capacity”* (476, veterinarian, UK).  *“Being asked to perform non-essential services by my employer”* (227, veterinarian, USA).  *“As a parent - having two kids at home whilst being deemed an 'essential worker' and working outside the home for more hours than ever - felt like the worse [sic] parent ever. As a veterinarian in a corporately owned small animal clinic - agreeing with the corporate we are an essential service but surely only for essential things. Corporate argued essential for everything even a routine desexing surgery or a nail trim. Felt I was undermining the efforts in the human pandemic by allowing people to travel to their vet for a trivial nail trim”* (465, veterinarian, Australia).  *“I am a regulatory vet and during the height of the pandemic in [identifier removed] the US was still importing and exporting horses, which are not an essential animal for food supply, etc. This put everyone from the vets, technicians, grooms, etc in contact with multiple people during each shipment, increasing everyone's risk of exposure to COVID, because we were considered "essential" personnel to keep the port open and facilitate trade”* (244, veterinarian, USA).  *“The most stressful challenge has been the question around spay/neuter. It was deemed a "non-essential" service by our state and all of the national professional animal welfare organizations said spay/neuter should be halted at the beginning of the pandemic. However, I work in an area where there is significant overpopulation of cats in particular. 3 months of no spay/neuter has been very stressful at the height of kitten season”* (85, veterinarian, USA). |
|  | Others failing to respect biosecurity | *“Clients challenging / complaining about the safety protocols we have put in place to both protect them and our team”* (264, veterinarian, Australia).  *“Clients who refuse to accept rulings around a lack of visitation to hospitalised patients - not sure how ethically challenging it is but it's certainly challenging emotionally and also to not lose compassion when they make our job harder through emotional blackmail techniques”* (209, veterinarian, Australia).  *“That some co-workers don't want to use facemask all the time, and sometimes we have to work very close, so we can either work alone, risk working with them or calling them to the administrations, knowing that they'll face problem, even send home (without pay), and we are already short staff because we are divided in shifts”* (185, veterinarian, Mexico).  *“working close together with colleagues that do not wear protection like face masks and therefore increase my risk to get infected (and this being announced as acceptable by my boss)”* (79, veterinarian, Germany). |
|  | Sickness presenteeism | *“Second-hand information of a colleague…with Covid-19 symptoms (not confirmed) who had reported for work and had not taken a COVID-19 test”* (21, veterinarian, Australia).  *“A team member becomes ill, tests positive for COVID and fails to disclose it to co-workers”* (451, veterinarian, USA).  *“Self-monitoring for symptoms and trying to make the decision of being on the safe side/staying home vs. coming into work as usual because you don't want to be an alarmist/create more work for peers by your absence”* (35, veterinary nurse, USA).  *“Developing upper respiratory symptoms & being concerned about notifying employers due to risk of losing job sooner (as a result of Covid - job was terminated)”* (300, veterinarian, Australia). |
| Client financial limitations | Financial limitations impacting standard of care | *“Client cannot afford optimal treatment so a "lesser" approach is chosen. Eg dispensing antibiotics when a stitch up would be in the animal's best interest”* (537, veterinarian, Australia).  *“Owners that are not able to pay for veterinary services due to loss of income and their pets are not able to obtain that standard of care that usually the owners are able to afford for them. Sometimes this means a patient receives suboptimal care (e.g. when hospitalization is recommended but owners are not able to afford during these times). This is tough as you know usually these clients would do anything for their pet yet as a practice there is a limit on how much credit u can extend to these clients”* (58, veterinarian, Singapore).  *“The most common ethically challenging issue is the inability of clients to afford treatment for their pets. This results in substandard treatments being used which prolong animal pain/illness…”* (409, veterinarian, Australia). |
|  | Euthanasia of animals with treatable conditions | *“Individuals who are unable to afford veterinary care due to losing their job during this pandemic - owners having to surrender their animals as they cannot afford care, or leaving medical issues for longer as they were unable to come in earlier…”* (135, veterinarian, Australia).  *“Euthanasia due to lack of finances secondary to loss of employment”* (490, veterinarian, Australia).  *“Increase in fixable cases being euth [sic]”*  (366, veterinary nurse, USA). |
| Animal welfare | End of life decision making | *“Being in a zoo environment, end-of-life decisions are made by a group; ethically it is challenging when team members do not always agree on the best course of action”* (446, veterinarian, USA).  *“End of life decisions”* (158, veterinarian, Canada).  *“Deciding on the right time for end of life in terms of balancing the welfare needs of the patient with the emotional needs of the client. The clients are more vulnerable at this challenging time of COVID-19, particularly our elderly clients living on their own who are isolated from their family and friends”* (172, veterinarian, Australia).  *“Decisions on euthanasia in wildlife cases”* (492, veterinarian, USA). |
|  | Euthanasia for objectionable reasons | *“Euthanasia of an animal who still got a good chance with treatment but owners won't bother (not money-related)”* (399, veterinarian, France).  *“Euthanasias of convenience”* (310, veterinarian, USA).  *“Culling healthy animals”* (250, animal health technician, Australia). |
|  | Futile treatment or euthanasia refusal | *“Client wishes to continue treatment when I consider it not in the best interest of the animal”* (231, veterinarian, Australia).  *“Persuading an animal owner that euthanasia is the best for the animal. Not seeing the animal enough due to Covid19 as a chronic condition and owner wanting just more pain relief”* (536, veterinarian, Australia).  *“Working with a rescue group who wants to spend a large amount of their time and resources on an untreatable animal's condition despite the likelihood it will continue to have a poor quality of life and be unable to find a suitable home”* (312, veterinarian, Australia).  *“Someone needing to euthanase their dog (for medical reasons - 100% necessary and the kindest thing for the dog...) and being quite reluctant to do so because it is their only companion in isolation”* (160, veterinarian, Australia). |
| Working conditions | Increased workload | *“EVERY DAY – The increase of phone calls, people demanding to see the vet, not having enough staff, managing the clients in the car park, keeping distancing managed, cleaning…long days, multi tasking -every day, no breaks”* (100, veterinary nurse, Australia).  *“Working at a ridiculous pace, to the extent of no breaks, 11-12 hour days & patient care compromised”* (367, veterinary nurse, Australia).  *“Insufficient resources to meet patient/client demands”* (249, veterinarian, UK).  *“With the increase in unemployment benefits and jobkeeper our clients have more money (and time) to access our services. The usual ethical issues are just as common. The new issue is that the significant increase in demand has exceeded our resources and animals are having to wait longer for treatment”* (383, veterinarian, Australia). |
|  | Reduced staff and/or services | *“We have had to limit services provided which results in ethically challenging situations”* (431, veterinarian, USA).  *“Staff being furloughed and replaced by higher paid administrative staff. These staff members then provided animal care, work in nutrition centre etc, often with no prior experience or having not performed this work in many cases over a decade”* (443, veterinarian, USA).  *“Balancing the needs of the hospital to have a teams based roster to minimise risk should someone contract COVID (so only half the team needs to isolate) with the needs of our staff to work full time or work certain days to receive the same shift loadings they normally would…”* (515, veterinarian, Australia). |
|  | Inability to provide appropriate level of care | *“Surge of clients wanting to be seen and vets taking on a much bigger daily workload. Mixed with less nurses working longer shifts leading to less individual care and attention time for each patient, higher chance of errors. Putting all patients at risk due to accepting nearly double the workload with half the staff”* (130, veterinary nurse, Australia).  *“Being so busy in the ER that we can’t properly take care of our patients while still charging clients a significant amount of money”* (293, veterinary nurse, Canada).  *“Cutting staffing by half and working in 2 teams but having no reduction in workload means that the smaller teams are overworked and pushed beyond capacity leading to compromised patient care”* (75, veterinary nurse, Australia). |
|  | Pressure to generate income | *“job threatened because I'm not producing enough revenue - told to charge more with no consideration to medical necessity”* (89, veterinarian, USA).  *“Balancing seeing as many cases as possible to ensure business revenue stayed as high as possible during a period of financial uncertainty with managing a caseload which prioritised patient care etc”* (462, veterinarian, Australia).  *“The constant push by the manager to bring in patients and not providing appropriate time to ensure proper evaluation”* (525, veterinary nurse, USA). |
|  | Team morale | *“Same as staff as per their comments I'm ruining their life as we put in restrictions if not allowing work gathering or food sharing or informing them to try not to socialise during the pandemic together”* (237, other animal health professional, Australia).  *“team stress and insecurity about jobs”* (221, veterinary nurse, Australia).  *“Having to tell the employees that my boss has decided that they have been laid off and will have no health insurance”* (4, other animal health professional, USA). |
| Client relations | Communication challenges | *“problems in communication (barriers, masks)”* (143, veterinarian, Germany).  *“Communication with clients is more stressful and is creating more ethically challenging situations. I am no longer able to give as much detail and it is more difficult to check for client understanding”* (7, veterinarian, Australia).  *“Lack of ability for owners to see what you have done (thoroughness of physical exam, proof of physical exam findings e.g. pain), lack of ability for me to visualise non-verbal signals of owners understanding/confusion of the disease process - all of which lead to frustrated owners, reduced treatment outcomes and complaints”* (466, veterinarian, Australia).  *“communication breakdown with a client that if they had been permitted to enter the building to see their -pet would not have resulted in the verbal confrontation and one star google review it did”* (228, veterinarian, Australia). |
|  | Mismatched expectations | *“Wishes and expectations of veterinarians versus clients”* (324, veterinarian, Australia).  *“The same issues as before COVID19. The mismatch of client expectations of vets to the ability of vets to provide an expected level of service for a perceived financial undertaking. Along with the mismatch of employee perceived self-worth for salaries, yet imposter syndrome making them not bill correctly. This is eternal”* (327, veterinarian, Australia).  *“Researchers who use animals demanded that animal supply was switched on immediately when the returned to work. This was demanded with no guarantee that all animals produced would be used. I had to engage in some very difficult conversations with researchers who are our customers”* (461, veterinarian, Australia). |
|  | Upset/aggressive/abusive clients | *“Abusive clients have increased…”* (319, veterinarian, Australia).  *“Public stress levels and explaining why they can’t be with their pet during a consult (emergency clinic)”* (341, veterinary nurse, Australia). |

**Supplementary Table 4.** Key themes identified in thematic analysis of free-text responses to online survey question “is there anything else you would like to add about your experience with ECS since the advent of COVID-19). N=195.

| **Theme** | **Sub-theme** | **Examples** |
| --- | --- | --- |
| The pandemic heightening anxiety/stress | Among people in general | *“Challenging client interactions have increased - the public seem less rational”* (534, veterinarian, Australia).  *“I feel the stress has provoked worse behaviour among a couple of my colleagues and employees and among my clients”* (229, veterinarian, China). |
|  | In veterinary settings | *“It has increased the amount of emotional, physical and mental stress. This has a negative effect on clinical decision making and the ability to decide the best course of action when faced with these ethically challenging situations”* (139, veterinarian, Australia).  *“COVID is a magnifying glass for all of the problems in the veterinary field... bad management, abusive owners, overwork, underpay, stress, burnout... all the problems are the same as before, they are just magnified”* (225, veterinarian, Australia). |
| The challenge of maintaining personal wellbeing | Difficulty accessing support networks | *“Balancing mental health away from work, without all the usual strategems available (personal interactions with friends/family; gym, dating)”* (99, veterinarian, Australia).  *“I missed my partner and my own pets. Worked a lot of unpaid hours. Had minimal time awake at home”* (475, veterinary nurse, New Zealand).  *“I've never felt so alone as in COVID 19”* (28, veterinarian, Canada). |
|  | Pandemic leading to stressors that impact personal wellbeing | *“Personally COVID caused me to become very burnt out due to increased euthanasia, increased stressed and rude clients and increased people who cannot treat their pets”* (269, veterinarian, Australia). |
| Veterinary teams and the veterinary professional strategies for managing in the pandemic situation | Teams navigated challenges well | *“…If anything, I have seen people being kinder and more flexible since COVID-19. We work hard at communication and clarity, and we just ramped it up”* (113, veterinarian, Canada).  *“We found the best strategy was to listen to everyone's concerns from the most productive vet down to the newest hospital assistant. Open communication was key. The most difficult part was the time limitation, decisions had to be made very quickly and there were no guidelines on how to do it right. Leadership through our governing body would have been helpful but we didn't have time to wait so used our best ethical judgement to make a call for staff and clients”* (511, veterinarian, Canada).  *“COVID itself I feel has been generally well managed in the veterinary practice situation”* (407, veterinarian, Australia). |
|  | Veterinary teams are well equipped to deal with pandemics | *“I think a lot of veterinarians have had to fall upon their public health training to make decisions for how to practice during this time, both to protect the staff members they work with and their clients/ visitors. I think we are uniquely trained to be able to make informed decisions to minimize disease transfer among those people (+/- animals) and am proud to be able to help make those decisions. Many business-owners in other segments of the population don't have this background, so it may be even more stressful for them given their lack of training in epidemiology“* (446, veterinarian, USA). |
